# Mislocalization of Nucleocytoplasmic Transport Proteins in Human Huntington’s Disease PSC-Derived Striatal Neurons

**DOI:** 10.3389/fncel.2021.742763

**Published:** 2021-09-29

**Authors:** Jenny Lange, Alison Wood-Kaczmar, Aneesa Ali, Sahar Farag, Rhia Ghosh, Jennifer Parker, Caroline Casey, Yumiko Uno, Akiyoshi Kunugi, Patrizia Ferretti, Ralph Andre, Sarah J. Tabrizi

**Affiliations:** ^1^Huntington’s Disease Centre, Department of Neurodegenerative Disease, UCL Queen Square Institute of Neurology, University College London, London, United Kingdom; ^2^Neuroscience Drug Discovery Unit, Takeda Pharmaceutical Company Limited, Fujisawa, Japan; ^3^Stem Cell and Regenerative Medicine Section, UCL Great Ormond Street Institute of Child Health, London, United Kingdom; ^4^UK Dementia Research Institute, University College London, London, United Kingdom

**Keywords:** Huntington’s disease, nuclear pore complex, striatal neurons, antisense oligonucleotide, pluripotent stem cell (PSC)

## Abstract

Huntington’s disease (HD) is an inherited neurodegenerative disorder caused by a CAG repeat expansion in the huntingtin gene (*HTT*). Disease progression is characterized by the loss of vulnerable neuronal populations within the striatum. A consistent phenotype across HD models is disruption of nucleocytoplasmic transport and nuclear pore complex (NPC) function. Here we demonstrate that high content imaging is a suitable method for detecting mislocalization of lamin-B1, RAN and RANGAP1 in striatal neuronal cultures thus allowing a robust, unbiased, highly powered approach to assay nuclear pore deficits. Furthermore, nuclear pore deficits extended to the selectively vulnerable DARPP32 + subpopulation neurons, but not to astrocytes. Striatal neuron cultures are further affected by changes in gene and protein expression of RAN, RANGAP1 and lamin-B1. Lowering total HTT using *HTT-*targeted anti-sense oligonucleotides partially restored gene expression, as well as subtly reducing mislocalization of proteins involved in nucleocytoplasmic transport. This suggests that mislocalization of RAN, RANGAP1 and lamin-B1 cannot be normalized by simply reducing expression of CAG-expanded HTT in the absence of healthy HTT protein.

## Introduction

Huntington’s disease (HD) is a progressive, invariably fatal neurodegenerative disorder characterized by choreic movements, psychomotor decline, dementia and psychiatric symptoms (Ross and Tabrizi, 2011; Tabrizi et al., 2020). It is caused by a CAG repeat expansion in exon 1 of the huntingtin (*HTT*) gene, which in unaffected individuals contains thirty five or fewer CAGs, whereas individuals carrying forty CAGs or more are certain to develop the disease (Andrew et al., 1993). Longer CAG expansions result in an earlier disease onset, and upwards of sixty results in juvenile onset HD. At the cellular level, HD is characterized by the early, selective neurodegeneration of striatal medium spiny neurons and cortical pyramidal neurons, whilst other neuronal populations are relatively unaffected (Rosas et al., 2008; Johnson et al., 2020). Despite ubiquitous expression of the mutant huntingtin protein (mHTT) and accumulation of aggregates (Davies et al., 1997; DiFiglia et al., 2016), the reasons for the early and selective loss of these neuronal subpopulations and the precise mechanisms by which mHTT causes neurodegeneration remain relatively unknown. Recent research has provided evidence that nuclear pore complex (NPC) function is disrupted in normal aging and dysfunction may contribute to neuronal loss in several neurodegenerative diseases, including frontotemporal dementia (FTD), amyotrophic lateral sclerosis (ALS), Parkinson’s disease (PD) and HD (Patel and Chu, 2011; Zhang et al., 2015; Grima et al., 2017; Chou et al., 2018).

NPCs span the double membranes of the nuclear envelope in eukaryotic cells and serve as the main conduit between the cell nucleus and cytoplasm. They are responsible for macromolecular trafficking of proteins between the two cellular compartments (Wente and Rout, 2010; Lin and Hoelz, 2019). Composed of approximately 30 nucleoporins (NUPs), which differ in functions including import and export, RNA export and membrane anchoring, NPCs are organized into five distinct regions, namely the central scaffold, cytoplasmic filaments, cytoplasmic and nuclear rings, the nuclear basket and the central pore channel (Alber et al., 2007). Molecules larger than 40kDA require active transport through the central channel of the NPC, which is facilitated by interactions of NUPs and nuclear transport receptors (Strambio-De-Castillia et al., 2010; Wente and Rout, 2010).

Active nucleocytoplasmic transport is facilitated by the small GTPase RAN, a GTP binding nuclear protein. RAN is crucial for both import and export, and is found primarily in the nucleus (D’Angelo et al., 2009; Raices and D’Angelo, 2012; Floch et al., 2014). During import, cargo is released when the transport-receptor interacts with RAN-GTP; during export, RANGAP1, a GTPase activating protein also involved in import, hydrolyzes RAN-GTP to generate RAN-GDP upon which cargo is released into the cytoplasm (Hetzer et al., 2002). RANGAP1 is located on the cytoplasmic filaments of the NPC and plays a crucial role in maintaining the nuclear/cytoplasmic (N/C) gradient of RAN (Matunis et al., 1996; Cole and Hammell, 1998). RANGAP1 is closely associated with RAN function and is required for bi-directional transport across the nuclear pore complex. Higher levels of RAN in the nucleus are crucial for maintaining active transport and loss of the N/C gradient has been shown to block nucleocytoplasmic transport (Mattaj and Englmeier, 1998).

Previous research has suggested that reduced nuclear export may be one of the causes of the pathogenic accumulation of mHTT in the nucleus (Truant et al., 2007; Kim and Taylor, 2017). In both HD mouse and human post-mortem brain tissue, several NUPs and RANGAP1 have been shown to co-localize with mHTT, and mHTT has been shown to exhibit preferential binding to RANGAP1 (Hosp et al., 2015). Defects in NPCs and nucleocytoplasmic transport proteins have been described in human HD and disease models (Gasset-Rosa et al., 2017; Grima et al., 2017; Alcalá-Vida et al., 2021). These included a shift of RAN-GTP, RANGAP1 and the nucleoporins NUP62 and NUP88 from the nucleus to cytoplasm. Reduction of insoluble mHTT in mouse brains lead to a reduction in insoluble RANGAP1 aggregates and overexpression of RANGAP1 or RAN alongside mHTT appeared neuroprotective and resulted in reduced cell death in transfected neurons (Grima et al., 2017).

In order to investigate nuclear pore complex dysfunction in human HD neurones and overcome limitations of previous studies, such as underpowered design and human bias in sample selection (Ketteler and Kriston-Vizi, 2016; Sherman and Bang, 2018), we developed a high content imaging approach. Mislocalization of proteins involved in nucleocytoplasmic transport was successfully detected using the Opera Phenix system for high content analysis in striatal neurones derived from isogenic embryonic stem cell lines (with 30, 45 and 81 polyQ repeat lengths) and a series of highly genetically related iPSC lines (with 22, 58, 69 and 75 polyQ repeat lengths). Given the lack of information on nuclear pore pathology in medium spiny neurons and astrocytes, we expanded our investigation to these cell types, and also investigated transcriptional changes and protein expression of nuclear pore transport components. Finally, we wished to establish whether the presence of healthy HTT is an absolute requirement for normal nucleocytoplasmic transport protein distribution, or this could be re-established in HD neurones by lowering total levels of diseased HTT using antisense oligonucleotides.

## Materials and Methods

### Cell Lines

Induced PSCs were generated from fibroblasts of skin biopsies taken from three siblings with juvenile Huntington’s disease, carrying *HTT* mutations with 56, 67 and 73 CAGs respectively, and their unaffected parent as a non-disease control. Biopsies were performed in accordance with the Declaration of Helsinki and approved by the University College London (UCL)/UCL Hospitals Joint Research Ethics Committee (LREC 03/N008, amendment 16). The subjects were recruited through the Huntington’s disease clinic at the National Hospital for Neurology and Neurosurgery, London; all subjects provided informed written consent. Two adjacent 3 mm punch biopsies taken from each subject’s forearm were cut into squares of 0.5–1 mm^2^ and placed epidermis side up into a well of a 6-well plate containing two drops of pre-warmed fibroblast culture medium (DMEM with glutamax, 4.5 g/L glucose and 1 mM pyruvate (Gibco), 10% fetal bovine serum, 50 U/ml penicillin, 50 μg/ml streptomycin, 2.5 ml/L amphotericin). A sterile coverslip was placed over the pieces of tissue to aid adherence to the plate and a further 2 ml pre-warmed medium was added to the well. Following incubation for 1 week at 37°C, 5% CO_2_, the media was changed; henceforth, cells were media changed every 3 to 4 days and passaged when required using 0.05% Trypsin-EDTA, reseeding each time at 1 × 10^4^ cells/cm^2^. During expansion of the cultures, cells were frozen for storage in liquid nitrogen. Induced PSCs were generated by Sendai virus reprogramming of the fibroblasts using the CytoTune-iPS 2.0 Sendai reprogramming kit (Thermo Fisher). These were validated by the expression of pluripotency markers, differentiation into all germ layers using a self-organisation assay, karyotyping, Sanger sequencing to confirm the *HTT* CAG repeat length and confirmation of the absence of exogenous Sendai virus. Two or three validated iPSC clones were generated from each subject. HTT polyglutamine (Q) lengths are expressed as *HTT* CAG(n)-length plus two (CAA, CAG) trinucleotides and herein the lines are referred to as the 22Q, 58Q, 69Q, or 75Q HD Family lines (Table 1). The IsoHD ESC lines comprising lines with 30Q, 45Q, and 81Q were generated and validated as described in Ooi et al. (2019) (Table 1).

**TABLE 1:** Control and Huntington’s disease cell lines.

Cell line	Cell type	Q length
IsoHD-30Q	ESC	30
IsoHD-45Q	ESC	45
IsoHD-81Q	ESC	81
HD-Family – 22Q	IPSC	22
HD-Family – 58Q	IPSC	58
HD-Family – 69Q	IPSC	69
HD-Family – 75Q	IPSC	75

### Cell Culture

Prior to neural induction, PSC lines were maintained in Essential 8 Media (Thermo Fisher Scientific) on Geltrex coated plates (1% in DMEM –F12, Thermo Fisher Scientific) and were passaged at approximately 80% confluency using RELESR (Stemcell Technologies) according to manufacturer’s instructions.

### Neural Induction and Differentiation of Human Pluripotent Stem Cells to Striatal Neurons

Neuronal differentiation followed the protocol (Arber et al., 2015) until day of differentiation (DD) 19, and was modified to allow high content imaging. Briefly, cells were maintained in N2B27 media (DMEM/F12 (67%), Neurobasal (33%), L-glutamine (1:100), N2 supplement (1:150), B27 supplement without vitamin A (1:150), β-mercaptoethanol (100 μM)) with SMAD inhibitors (LDN193189 (100 nM), SB431542 (10 μM) and dorsomorphin (200 nM)) until DD9, after which they were changed into N2B27 media containing activin A (25 ng/ml). On DD19-20 a second passage onto laminin coated 6 well plates was performed using 0.02% EDTA and media was changed on DD26 to MSN media (N2B27 media with B27 (vitamin A), activin A, penicillin/streptomycin, BDNF and GDNF). Neuronal cultures that were to be used for high content imaging were passaged again using Accutase on DD28 and plated onto Geltrex coated PerkinElmer CellCarrier-96 well microplates at a density of 4 × 10^4^ cells/well. Three differentiations were completed per experiment (technical replicates), per clone where there was more than one available.

### Differentiation of Human Pluripotent Stem Cells to Astrocytes

Astrocytes were differentiated as described previously (FitzPatrick et al., 2018). Briefly, PSCs were plated at 1 × 10^4^ cells per well in u-bottom 96-well plates in neural induction medium with ROCK inhibitor for 24 h, after which neuroepithelial clusters became visible and a full media change with neural induction medium without ROCK inhibitor was completed daily for 5 days. Clusters were then plated onto Laminin coated 6-well plates and neural rosettes formed after a further 4 days. These were manually picked, centrifuged at 200 *g* for 5 min and re-plated into Laminin coated 6 well plates in neural maintenance medium. Neural progenitor like cells were maintained for 5 passages before being plated into astrocyte medium to induce differentiation. Astrocytes were maintained for 3 weeks and then plated into 96-well plates at 48 × 10^4^ cells per plate. After a further 48 h astrocytes were fixed in 4% PFA and kept at 4 C until immunostaining was completed.

### Immunostaining

Neuronal cultures in 96 well plates were fixed on DD 37 with 4% paraformaldehyde at room temperature for 15 min, washed three times in PBS and stored at 4°C in PBS containing 0.02% sodium azide. Fixed cells were permeabilized with 0.02% Triton for 15 min, washed with PBS, incubated for 1 h in blocking solution (10% goat serum, 1% BSA in PBS) and finally incubated with primary antibodies (Table 2) in PBS containing 1% BSA overnight at 4°C. Primary antibodies were washed off the next day 5 times with PBS after which cells were incubated for 1 h at room temperature with Alexa Fluor conjugated secondary antibodies (1:1000) and Hoechst (1:500, 1 μg/ml) in PBS containing 1% BSA. After a further 5 washes in PBS, fixed cells were kept in PBS containing 0.02% sodium azide at 4°C until imaging. Staining for lines that were to be directly compared was carried out at the same time to allow for comparison of staining intensity.

**TABLE 2:** Antibodies used for immunostaining.

Antibody	Manufacturer	Catalog number	Dilution
MAP2	Abcam	Ab32454	1:1000
βIIItubulin	Abcam	Ab78078 or Ab107216	1:500
DARPP32	Abcam	Ab40802	1:200
CTIP2	Abcam	Ab18465	1:250
RAN	Insight Biotechnology	SC-271376	1:250
RANGAP1	Insight Biotechnology	SC-28322	1:250
lamin-B1	Abcam	Ab16048	1:500
NUP62	Insight Biotechnology	SC-48389	1:200
NUP88	Abcam	Ab79785	1:200
EM48	Millipore	MAB2166	1:100

### High Content Imaging

All high content imaging was carried out using the Opera Phenix high content screening system (PerkinElmer Cellular Technologies) with a Harmony user interface software. Cells stained for neuronal markers (MAP2, DARPP32, CTIP2) and aggregate markers (EM-48, S830) were imaged using a confocal 40× air 0.6 objective with a correction collar of 0.18 mm. Neurons stained with nuclear pore markers (Table 1) and neuronal markers were imaged using a confocal 63× air objective. Binning for both conditions was set at 2 and twenty fields of view in each of six replicate wells were imaged per condition, using the Hoechst 33342, 488 nm and 568 nm channels. This allowed us to image a minimum of 1500 MAP2 + neurons per experiment (*n* = 1), thus examining at least 4500 neurons across *n* = 3 biological samples.

Well set height for neuronal cultures was set at 5 μm (40× objective) or 2 μm (63× objective), which was initially optimized by generating a Z-stack for each condition. Astrocytes stained for GFAP and Vimentin were imaged using a 20× air objective with a correction collar of 0.19 mm. Astrocytes stained with nuclear pore markers (Table 1) and neuronal markers were imaged using a confocal 63× air objective. Binning was set at 2 and twenty fields of view in each of six replicate wells were imaged per condition, using the channels Hoechst 33342, 488 nm, 568 nm, and 647 nm channels. Well set height for astrocyte cultures was set at 1 μm, which was initially optimized by generating a Z-stack for each condition.

### Image Processing

Columbus image software (v2.8.0, Perkin Elmer) was used to analyze and quantify images by creating a script for each condition on the relevant control line (IsoHD 30Q or 22Q) before running a batch analysis on the matching HD lines (Tables 3–5; Supplementary Methods). Briefly, we obtained mean staining intensity for nuclear pore markers (Table 1) in nucleus and cytoplasm of MAP2 positive neurons (Supplementary Figure 2). We divided mean staining intensity in the nucleus by mean staining intensity in the cytoplasm (Supplementary Figure 3) to obtain a mean intensity nuclear/cytoplasmic ratio of nuclear pore proteins. For astrocytes, we obtained mean staining intensity for nuclear pore markers in nucleus and cytoplasm of vimentin or GFAP positive astrocytes. Results were exported to Excel and analyzed in GraphPad Prism8.

**TABLE 3:** Perkin Elmer Columbus System parameters for script analysis (neuronal culture composition and mutant huntingtin aggregation).

Cell feature	Columbus building block	Specifics	Method	Output name
Nuclei	Find Nuclei	Channel: HOECHST 33342 Region: None	Method: M Diameter: 15 μm Splitting Sensitivity: 0.4 Common threshold: 0.1	Population: Nuclei
	Calculate Intensity Properties	Channel: HOECHST 33342 Population: Nuclei Region: Nucleus	Method: Standard, mean	Properties: Intensity nuclear HOECHST 33342
	Calculate Morphology Properties	Population: Nuclei Region: Nucleus	Method: Standard, area, roundness	Properties: Nucleus morphology
	Select Population	Population: Nuclei	Method: Filter by property Nucleus area [μm^2^]: > = 40 Intensity nucleus HOECHST 33342 mean: < = 18512 Nucleus area [μm^2^]: < = 250 Boolean operations: F1 and F2 and F3	Population: Viable nuclei
BIII-tubulin +	Find Cytoplasm	Channel: Alexa 633 Nuclei: Viable nuclei	Method: B Common threshold: 0.45 Individual threshold: 0.15	
	Calculate Intensity Properties	Channel: Alexa 633 Population: Viable nuclei Region: Cytoplasm	Method: Standard, mean	Properties: Intensity cytoplasm Alexa 647
	Select Population	Population: Viable nuclei	Method: Filter by Property Intensity cytoplasm Alexa 647, mean: > = 1800	Population: Viable nuclei with cytoplasmic B3 tubulin over a threshold
CTIP2 +	Calculate Intensity Properties	Channel: Alexa 568 Population: Viable nuclei Region: Nucleus	Method: Standard, mean	Properties: Intensity nucleus Alexa 568
	Select Population	Population: Viable nuclei	Method: Filter by property Intensity nucleus Alexa 568 mean: > = 500	Population: CTIP + cells
S830 + spots	Find Spots	Channel: Alexa 488 Region: Viable nuclei	Method: B Detection sensitivity: 0.05 Splitting coefficient: 1 Calculate spot properties	Population: S830 + spots in viable nuclei
	Select Population	Population: Viable nuclei	Method: Filter by property Number of spots: > 4	Population: Viable nuclei with more than four spots
EM48 + spots	Find Spots	Channel: Alexa 568 Region: Viable nuclei	Method: A Relative spot intensity: > 0.045 Splitting coefficient: 0.995 Calculate spot properties	Population: EM48 + spots in viable nuclei
	Select Population	Population: Viable nuclei	Method: Filter by property Number of spots: > 4	Population: Viable nuclei with > 4 EM48 + spots
DARPP32 + neurons	Find Cells	Channel: Alexa 488 Region: None	Method: B Common threshold: 0.4 Area: > 70 μm^2^ Split Factor: 7 Individual threshold: 0.5 Contrast: > 0.3	Population: DARPP32 + cells (Find Cells)

**TABLE 4:** Perkin Elmer Columbus System parameters for script analysis (neuronal cultures and nuclear pore staining).

Cell feature	Columbus building block	Specifics	Method	Output name
Nuclei	Find Nuclei	Channel: Hoechst 33342	Method: M Diameter: 15 μm Common Threshold: 0.1	Population: Nuclei
	Calculate Intensity Properties	Channel: Hoechst 33342 Population:Nuclei Region: Nucleus	Method: Standard, mean	Properties: Intensity nucleus Hoechst 33342
	Calculate Morphology Properties	Population:Nuclei Region: Nucleus	Method: Standard, area, roundness	Properties: Nucleus Morphology
	Select Population	Population: Nuclei	Method: Filter by property Nucleus area (μm^2^): > 40 Nucleus area (μm^2^): < 200 Intensity nucleus Hoechst 33342 mean: < 18512 Nucleus roundness: > 0.4	Population: Viable nuclei
MAP2 + Cells	Find Cytoplasm	Channel: Alexa 633 Nuclei: Viable Nuclei	Method:B Common threshold: 0.45 Individual threshold: 0.15	
	Calculate Intensity Properties	Channel: Alexa 633 Population: Viable nuclei Region: Nucleus	Method: Standard, mean	Properties: MAP2 intensity in nucleus area (Alexa 647)
	Calculate Intensity properties	Channel: Alexa 633 Population: Viable nuclei Region: Cytoplasm	Method: Standard, mean	Properties: MAP2 intensity in cytoplasmic area (Alexa 647)
RAN + cells	Calculate Intensity Properties	Channel: Alexa 488 or 594 Population: Viable nuclei Region: Nucleus	Method: Standard, mean	Properties: RAN intensity in nucleus area (Alexa 488 or 594)
	Calculate Intensity properties	Channel: Alexa 488 or 594 Population: Viable nuclei Region: Cytoplasm	Method: Standard, mean	Properties: RAN intensity in cytoplasmic area (Alexa 488 or 594)
RANGAP1 + cells	Calculate Intensity Properties	Channel: Alexa 488 or 594 Population: Viable nuclei Region: Nucleus	Method: Standard, mean	Properties: RANGAP1 intensity in nucleus area (Alexa 488 or 594)
	Calculate Intensity properties	Channel: Alexa 488 or 594 Population: Viable nuclei Region: Cytoplasm	Method: Standard, mean	Properties: RANGAP1 intensity in cytoplasmic area (Alexa 488 or 594)
lamin-B1 + cells	Calculate Intensity Properties	Channel: Alexa 488 or 594 Population: Viable nuclei Region: Nucleus	Method: Standard, mean	Properties: lamin-B1 intensity in nucleus area (Alexa 488 or 594)
	Calculate Intensity properties	Channel: Alexa 488 or 594 Population: Viable nuclei Region: Cytoplasm	Method: Standard, mean	Properties: lamin-B1 intensity in cytoplasmic area (Alexa 488 or 594)
NUP62 + cells	Calculate Intensity Properties	Channel: Alexa 488 or 594 Population: Viable nuclei Region: Nucleus	Method: Standard, mean	Properties: NUP62 intensity in nucleus area (Alexa 488 or 594)
	Calculate Intensity properties	Channel: Alexa 488 or 594 Population: Viable nuclei Region: Cytoplasm	Method: Standard, mean	Properties: NUP62 intensity in cytoplasmic area (Alexa 488 or 594)
Nup88 + cells	Calculate Intensity Properties	Channel: Alexa 488 or 594 Population: Viable nuclei Region: Nucleus	Method: Standard, mean	Properties: NUP88 intensity in nucleus area (Alexa 488 or 594)
	Calculate Intensity properties	Channel: Alexa 488 or 594 Population: Viable nuclei Region: Cytoplasm	Method: Standard, mean	Properties: NUP88 intensity in cytoplasmic area (Alexa 488 or 594)

**TABLE 5:** Perkin Elmer Columbus System parameters for script analysis (astrocyte cultures).

Cell feature	Columbus building block	Specifics	Method	Output name
Nuclei	Find Nuclei	Channel: Hoechst 33342	Method: B Diameter: > 30 μm Common Threshold: 0.4	Population: Nuclei
	Calculate Intensity Properties	Channel: Hoechst 33342 Population:Nuclei Region: Nucleus	Method: Standard, mean	Properties: Intensity nucleus Hoechst 33342
	Calculate Morphology Properties	Population:Nuclei Region: Nucleus	Method: Standard, area, roundness	Properties: Nucleus Morphology
	Select Population	Population: Nuclei	Method: Filter by property Nucleus area (μm^2^): > 40 Nucleus area (μm^2^): < 1000 Intensity nucleus Hoechst 33342 mean: < 18512 Nucleus roundness: > 0.4	Population: Viable nuclei
Vimentin + cells	Find Cytoplasm	Channel: Alexa 488 Nuclei: Viable Nuclei	Method:A Individual threshold: 0.15	
GFAP + cells	Find Cytoplasm	Channel: Alexa 633 Nuclei: Viable Nuclei	Method:A Individual threshold: 0.15	
RAN + cells	Calculate Intensity Properties	Channel: Alexa 488 Population: Viable nuclei Region: Nucleus	Method: Standard, mean	Properties: RAN intensity in nucleus area (Alexa 488)
	Calculate Intensity properties	Channel: Alexa 488 Population: Viable nuclei Region: Cytoplasm	Method: Standard, mean	Properties: RAN intensity in cytoplasmic area (Alexa 488)
RANGAP1 + cells	Calculate Intensity Properties	Channel: Alexa 488 Population: Viable nuclei Region: Nucleus	Method: Standard, mean	Properties: RANGAP1 intensity in nucleus area (Alexa 488)
	Calculate Intensity properties	Channel: Alexa 488 Population: Viable nuclei Region: Cytoplasm	Method: Standard, mean	Properties: RANGAP1 intensity in cytoplasmic area (Alexa 488)
lamin-B1 + cells	Calculate Intensity Properties	Channel: Alexa 594 Population: Viable nuclei Region: Nucleus	Method: Standard, mean	Properties: lamin-B1 intensity in nucleus area (Alexa 594)
	Calculate Intensity properties	Channel: Alexa 594 Population: Viable nuclei Region: Cytoplasm	Method: Standard, mean	Properties: lamin-B1 intensity in cytoplasmic area (Alexa 594)

### Western Blotting

Protein was extracted from cell pellets obtained from neuronal cultures at DD37 of three 22Q clones and three 75Q clones. These were mechanically disassociated in RIPA buffer (Sigma) supplemented with Complete protease inhibitors (Roche). Cell lysates were kept at 4°C on ice for 30 min, then centrifuged for 15 min at 10,000 *g* (at 4°C). Protein concentrations were measured using a BCA kit (Thermo Fisher Scientific) according to manufacturer’s instructions. Samples were kept at −20°C until ready to be used for Western blotting.

Samples were denatured in BOLT LDS sample buffer and reducing agent (Thermo Fisher Scientific) for 10 min at 70°C. Fifty micrograms of total protein were loaded per lane in 4–12% Tris-Bis Plus^TM^ gels (Thermo Fisher Scientific) in a Xcell Sure Lock Mini Cell (Thermo Fisher Scientific). Twelve μl Bio Rad Precision Plus Protein All Blue Standards were loaded as a size reference. All gels were run in MOPS running buffer at 220V, except for gels containing samples where RAN levels were to be measured and MES running buffer was used. Proteins were transferred onto a 0.2 μm nitrocellulose membrane (BioRad) in BOLT transfer buffer (Thermo Fisher Scientific) at 35 V for two and a half hours at 4°C. After confirming transfer using Ponceau red staining, membranes were incubated at room temperature in Li-Cor Interceptor Buffer for 1 h. Incubation with primary antibodies (Table 6) diluted in Li-Cor interceptor buffer was carried out overnight at 4°C, after which samples were washed 3 times in TBS-T (0.02% Tween) for 10 min. Li-Cor conjugated secondary antibodies (700 nm and 800 nm) were diluted 1:5000 in Li-Cor Interceptor buffer and membranes were incubated at room temperature for a further hour. After three washes in TBS-T and three washes in TBS, membranes were imaged on an Odyssey System. Band signal intensity was measured using Image Studio 5.2 software (Li-Cor) and normalized to that of β-actin; measurements were exported into Excel and analyzed in GraphPad Prism 8. A minimum of three technical repeats were measured per 75Q and 22Q clone from the HD Family lines and 30Q, 45Q, and 81Q for the IsoHD lines.

**TABLE 6:** Antibodies used for Western Blotting.

Antibody	Manufacturer	Catalog number	Dilution
Actin	Sigma	A5316	1:1000
RAN	Abcam	ab155103	1:100
RANGAP1	Abcam	ab92360	1:100
lamin-B1	Abcam	ab16048	1:200
Huntingtin	Millipore	Mab2166	1:1000

### Anti-HTT Antisense Oligonucleotides Delivery

Antisense oligonucleotides (ASO) to exon 36 of HTT (5′-CTCAGtaacattgacACCAC-3′) (Kordasiewicz et al., 2012) and nanoparticles were provided by Takeda Pharmaceuticals. ASOs were prepared in Buffer A to a concentration of 4 μM and added to nanoparticles diluted prior in 0.9% saline to a concentration of 0.853 mM. The ASO-nanoparticle complex was then diluted further in the appropriate culture medium to a final concentration of 30 nM ASOs. ASOs were delivered after Passage 2 on DD 20 to HD Family 22Q (3 clones), HD Family 75Q (3 clones), IsoHD 30Q and IsoHD 81Q (3 differentiations). Media changes with the ASO-nanoparticle complex were completed every 2–3 days and pellets for qPCR analysis were taken on DD 37.

### Quantitative PCR

Cells at DD 37 were centrifuged and pellets washed twice in PBS, before being snap frozen and stored at −20°C until further use. RNA was extracted using an RNEasy minikit (Qiagen) according to manufacturer’s instructions. cDNA was synthesized using Superscript IV (Thermo Fisher Scientific) adhering to the manufacturer’s instructions. Briefly, 2 μg RNA was added to 1 μl dNTPs (10 mM) and 1 μl random hexamers (100 ng/μl) and topped up to 13 μl with RNAse free water. Samples were heated at 65°C for 5 min, then chilled on ice for 1 min before adding 4 μl 5× Superscript IV buffer, 1 μl Superperscript IV Reverse Transcriptase, 1 μl DTT (100 mM) and 1 μl RNAse Inhibitor per reaction. Samples were cycled for 10 min at 23°C, 55°C and 80°C after which 1 μl RNAse H was added to each sample and incubated for 20 min at 37°C.

Samples were either kept at 4°C or were frozen until qPCR could be performed. Next, 3.5 μl sample was added to 3.75 μl water, 7.5 μl TaqMan Fast Advanced Mastermix (Thermo Fisher Scientific) and 0.75 μl of HTT probe (Table 7) and housekeeper genes (ATP5B, UBC, EIF4; Thermo Fisher Scientific) in a 96 well plate and quantified in a QuantStudio 5 ProFlex system using the following conditions: 95°C for 40s, 40 cycles of 95°C for 15 s and 60°C for 30 s. Data obtained were analyzed using the comparative cycle threshold method (ΔΔCt) (Schmittgen and Livak, 2008).

**TABLE 7:** Taqman (Thermofisher) qPCR probes.

Target	Catalog number	Fluorophore
*EIF4A2*	Hs00756996_g1	VIC/FAM
*ATP5B*	Hs00969569_m1	VIC
*UBC*	Hs05002522_g1	VIC
*HTT*	Hs00918174_m1	FAM
*RAN*	Hs03044733_g1	FAM
*RANGAP1*	Hs00610049_m1	FAM
*Lamin-B1*	Hs01059210_m1	FAM

### Quantification and Statistical Analysis

For all quantification, each IsoHD line was differentiated three times in independent experiments. For HD Family lines where three clones were available (22Q, 75Q), each of these were differentiated, for all other HD Family lines (58Q and 69Q) a minimum of three separate differentiations were carried out.

All statistical analysis was carried out in Graphpad Prism 8 and a Student’s *t*-test or one way ANOVA with Bonferroni’s multiple comparisons correction was carried out between isogenic lines with a 95% confidence interval and values with *p* < 0.05 were considered statistically significant. Statistical significance is indicated as ^∗^: *p* < 0.05, ^∗∗^: *p* < 0.01, ^∗∗∗^: *p* < 0.001, ^****^: *p* < 0.0001. All data is reported at mean ± SEM.

## Results

### High Content Imaging of Human HD Pluripotent Stem Cells Differentiated to a Striatal Neuron Fate

Genetic backgrounds of PSC donors are known to influence transcription and differentiation of the resulting cell lines (Rouhani et al., 2014; Burrows et al., 2016; Kyttälä et al., 2016), making direct comparisons between different PSC lines difficult (Germain and Testa, 2017). In order to reduce such variability, we used iPSC lines obtained from an unaffected mother and her HD affected family (with polyQ lengths of 22Q, 58Q, 69Q, and 75Q) and differentiated these to striatal neurons, alongside lines of an isogenic ESC series (IsoHD, with lengths of 30Q, 45Q, and 81Q) (Ooi et al., 2019). Increasing CAG lengths in *HTT* exon 1 were incorporated using transcription activator-like effector nucleases in an extensively studied H9 hESC line to generate the IsoHD series and have been validated as described by Ooi et al. (2019). The HD family iPSC lines were generated from fibroblasts obtained from three siblings with juvenile HD and their unaffected parent. Induced PSCs were generated by Sendai virus reprogramming by Ali Brinvalou (Rockefeller University).

**FIGURE 1:**
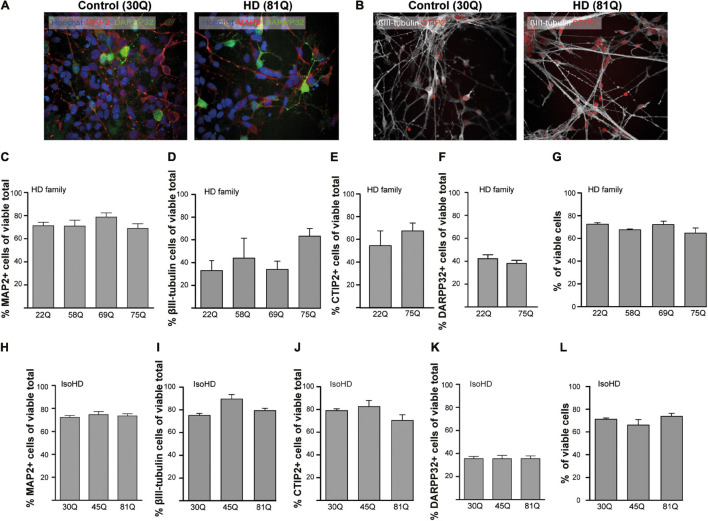
High content imaging of human HD PSCs differentiated to a striatal neuron fate. **(A)** All PSC lines used in the study generated DARPP32 + /MAP2 + and **(B)** CTIP + /βIII-tubulin + cells at day 37 differentiation, as shown in the example images, indicative of GABAergic striatal neurons. High content imaging was used to quantify the proportions of cells expressing these markers. Scale bar = 50 μM. **(C)** The proportion of cells expressing the mature, pan-neuronal marker, MAP2, showed no significant difference between HD Family line cell cultures expressing mutant HTT and those that do not. **(D)** The proportion of cells expressing the pan-early neuronal marker βIII-tubulin was more variable but no significant differences were detected between unaffected and HD Family line neuronal cultures. The expression of markers of a striatal medium spiny neuron fate were assessed, showing no significant differences in the proportions of cells expressing either **(E)** CTIP2 or **(F)** DARPP32 between the control and mutant HTT expressing cultures generated with the HD Family series. **(G)** Viability was calculated by quantifying numbers of pyknotic or otherwise dysmorphic nuclei as an inverse proportion of the total number of cells. This proportion was similar (∼70%) in all lines of HD Family line neuronal cultures. **(H)** No significant differences were detected in the proportion of Map2 + cells in the IsoHD series, nor in the proportion of cells expressing **(I)** βIII-tubulin, **(J)** CTIP2 or **(K)** DARPP32. **(L)** Viability was not affected in IsoHD neurons. Data are represented as mean ± SEM of at least one differentiation of each clone of the HD Family iPSC lines, or three differentiations of each of the IsoHD ESC lines, subject to one-way ANOVA followed by Bonferroni *post hoc* test or unpaired *t*-test with Welsh’s correction where appropriate.

PSC lines were differentiated to a striatal neuron fate using an activin A-based method of inducing lateral ganglionic eminence-like characteristics in neural progenitor cells (Arber et al., 2015). To characterize the cells in culture, a high content immunofluorescent imaging platform was chosen (Perkin Elmer Opera Phenix), which allows the rapid acquisition of high numbers of images without any user bias in the chosen fields-of-view. Each of the PSC lines used in the study generated neuronal cultures with the majority of cells staining positively for the pan-neuronal marker MAP2 (Figures 1A,C,H) at DD37 and exhibiting similar morphology. A subset of neurons expressed the pan-early neuronal marker βIII-tubulin though this was more variable between the IPSC-derived HD Family and ESC-derived IsoHD lines (Figures 1B,D,I), potentially indicating greater maturity of the HD Family line neurons. 60–70% of cells expressed CTIP2 + indicative of a GABAergic striatal fate (Figures 1B,E,J) and between 35 and 45% of neurons expressed DARPP32, a marker for medium spiny neurons (MSNs) (Figures 1F,K). No significant differences were detected between HD and unaffected lines. Around 20% of cells were stained positive for proliferation marker Ki67 with no significant differences between IPSC- or ESC- derived cultures or between control and HD (Supplementary Figure 1A), suggesting that the MAP2 negative cells are of neural progenitor or glial origin. We further stained cultures for neural progenitor marker Nestin, which stained 5–8% of cells, with no significant differences between control or HD cultures (Supplementary Figure 1B).

The canonical marker of HD pathology is the formation of HTT aggregates. Using established antibodies for the detection of aggregated HTT, S830 and EM48 (Bayram-Weston et al., 2016), we sought to examine whether HTT inclusions might be forming in HD striatal neurons. This revealed no significant differences between the lines above an apparent ‘background’ level of S830 + or EM48 + staining nuclear puncta (Supplementary Figure 2). Finally, given that all measures were quantified only in cells determined to be viable, it was important to determine whether there were any overt HTT polyQ-dependent effects on cell viability as previous studies in IPSC derived neurons have reported conflicting results (Mattis et al., 2012; Chiu et al., 2015; Song et al., 2018). This showed no significant differences in viability under basal conditions between control and HD lines in these PSC-derived striatal neuron cultures (Figures 1G,L) at DD37.

### Analysis of Nuclear Pore Phenotypes in HD Striatal Neurons Using High Content Imaging

Next, we wanted to investigate whether high content imaging would provide a suitable platform to detect aberrations in the localization of proteins associated with nucleocytoplasmic transport. We focused on RAN, RANGAP1 and lamin-B1, which have previously been shown to mislocalize in several HD models (Gasset-Rosa et al., 2017; Grima et al., 2017; Veldman and Yang, 2017; Alcalá-Vida et al., 2021).

**FIGURE 2:**
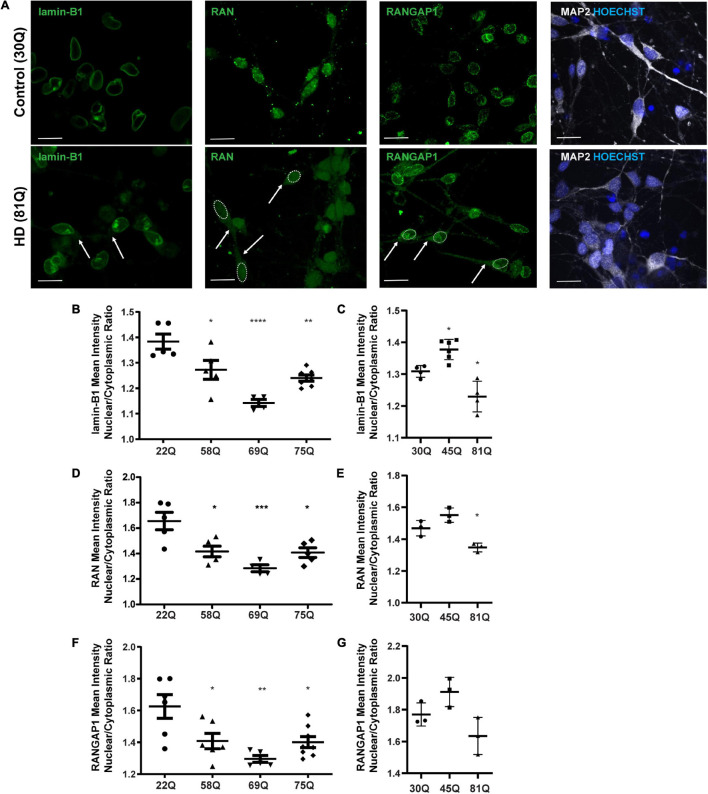
High content imaging can detect mislocalization of lamin-B1, RAN and RANGAP1 in human HD PSC-derived striatal neurons. **(A)** Neuronal cultures were stained for lamin-B1, RAN and RANGAP1 and were counterstained with Hoechst and MAP2. In HD neurons, lamin-B1, RAN and RANGAP1 are detected much more widely in soma and processes indicated by the arrows and outline of the nuclear area, compared to control cultures where expression is highly localized to the nucleus. Scale bar = 20 μM. **(B)** HD Family line 58Q, 69Q, and 75Q neurons exhibited significantly lower lamin-B1 mean intensity nuclear cytoplasmic (N/C) ratios than control 22Q neurons **(C)** The N/C ratio was significantly lower in IsoHD 81Q, compared to their respective control (IsoHD 30Q). **(D)** The RAN N/C ratio of the HD Family line 58Q, 69Q, and 75Q neurons was significantly lower than in control 22Q neurons, **(E)** as was the N/C ratio in IsoHD 81Q neurons compared to in IsoHD 30Q neurons, confirming that the RAN gradient is altered and RAN is mislocalized. **(F)** Neurons from the HD Family line 58Q, 69Q, and 75Q cultures all exhibited lower RANGAP1 N/C ratios than their respective control. **(G)** No significant differences were detected in the N/C ratio between control and IsoHD45Q or IsoHD 81Q neurons. *: *p* < 0.05, **: *p* < 0.01, ***: *p* < 0.001, ****: *p* < 0.0001. Data are represented as mean ± SEM of at least one differentiation of each clone of the HD Family iPSC lines, or three differentiations of each of the IsoHD ESC lines, subject to one-way ANOVA followed by Bonferroni *post hoc* test.

Here, the nuclear to cytoplasmic gradient of nuclear pore transport proteins is expressed as a nuclear/cytoplasmic (N/C) ratio of mean staining intensities within each compartment of MAP2 positive cells (Figure 2A and Supplementary Figure 3) at DD37. The absolute mean staining intensities for each compartment are presented in Supplementary Figure 4. No overt neuronal morphological phenotypes were detected between control and HD neurons, that may account for differential distribution of nuclear pore transport associated proteins (Supplementary Figure 5).

For the purpose of analysis, the nuclear lamina are considered as part of the nucleus, whereas any staining that does not co-localize with the nucleus is considered cytoplasmic. In the HD Family iPSC lines, the lamin-B1 N/C ratio was significantly lower in striatal MAP2 + neurons of all HD lines (58Q, 69Q, and 75Q) than the 22Q control (Figure 2B). No polyQ-length dependent changes were detected. In the ESC-derived IsoHD lines, the lamin-B1 N/C ratio was significantly lower in IsoHD 81Q neurons than the IsoHD 30Q neurons (Figure 2C). Interestingly, the lamin-B1 N/C ratio was significantly higher in IsoHD 45Q than in the control neurons, IsoHD 30Q, which may represent a compensatory mechanism in shorter polyQ-length striatal neurons.

In the HD Family series, we observed a significant reduction in the mean RAN N/C ratio in MAP2 positive neurons derived from each of the HD Family iPSC lines (58Q, 69Q, and 75Q) as compared to their respective non-disease control line (22Q) (Figure 2D). In neurons derived from the IsoHD ESC lines, the N/C ratio in IsoHD 81Q neurons was significantly lower than in IsoHD 30Q neurons (Figure 2E). No significant changes were apparent in the IsoHD 45Q neurons which could be a reflection of the shorter polyQ length of this line, potentially indicating that they are less vulnerable to nuclear pore defects at this stage. In both HD Family and IsoHD MAP2 positive neurons, RAN nuclear staining intensity was comparable between control and HD (Supplementary Figure 4A), whereas increased cytoplasmic intensity was apparent in 58Q, 69Q, 75Q, and IsoHD 81Q compared to control lines (Supplementary Figure 4B) suggesting an increased concentration of RAN within the cytoplasm. Although we observed RAN positive puncta (Figure 2A), there was no significant difference in percentage of neurons containing RAN puncta between HD and control neurons either in the nucleus or cytoplasm (Supplementary Figure 6). Similarly, we did not observe any evidence of RANGAP1 aggregation (Figure 2A).

RANGAP1 is tightly associated with the nuclear pore complex and our staining shows a tight ring around the outer nucleus. For the purposes of analysis, we consider this staining as nuclear (Figure 2A). In the HD Family series, we identified a significant reduction in the N/C ratio between the control 22Q and the 58Q, 69Q, and 75Q MAP2 positive neurons (Figure 2F). In the IsoHD lines, although a trend toward reduction of the N/C ratio was observed in the 81Q neurones, it did not reach statistical significance (Figure 2G).

Conversely, nucleoporin 62 and 88 mislocalization which has previously been reported (Grima et al., 2017), was detected in IsoHD 81Q neurons, but not in the IsoHD 45Q or the Family HD lines (Supplementary Figure 7). This may reflect differences in the genetic background of the iPSC lines, or in epigenetic changes, that modify the penetrance of nuclear pore defects or indicate that these nucleoporins are not universally disrupted across HD neurons.

In summary, high content imagining was sensitive enough to detect changes in RAN, RANGAP1 and lamin-B1 localization, and confirmed the presence of nuclear pore transport phenotypes in an isogenic HD line series as well as in an iPSC series with more genetic variation.

### Defects in Nuclear Pore Transport Proteins RAN and RANGAP1 Are More Pronounced in Medium Spiny Neurons

**FIGURE 3:**
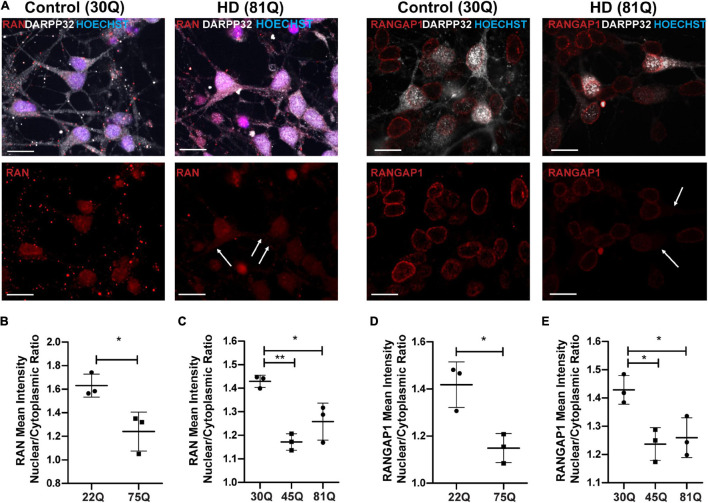
RAN and RANGAP1 are mislocalized in HD PSC-derived medium spiny neurons. Neuronal cultures were stained for MAP2 and DARPP32 to identify medium spiny neurons (MSNs), RAN and RANGAP1 and were counterstained with Hoechst. **(A)** Staining for RAN and RANGAP1 was more diffuse in HD MSNs than in control neurons, with greater staining in the soma as indicated by the arrows. Scale bar = 20 μM. **(B)** The RAN mean intensity nuclear cytoplasmic (N/C) ratio was significantly lower in 75Q DARPP32 + /MAP2 + MSNs than in 22Q MSNs. **(C)** The RAN N/C was also significantly lower in IsoHD 45Q and IsoHD 81Q MSNs than their respective control (IsoHD 30Q). When we assessed the RANGAP1 N/C ratio specifically in DARPP32 + /MAP2 + MSNs we found a significantly lower ratio across all HD lines **(D)** HD Family 75Q vs. 22Q, **(E)** IsoHD 45Q and IsoHD 81Q vs. IsoHD 30Q) *: *p* < 0.05, **: *p* < 0.01. Data are presented as mean ± SEM of at least one differentiation of each clone of the HD Family iPSC lines, or three differentiations of each of the IsoHD ESC lines, analyzed by one-way ANOVA with Bonferroni correction or unpaired *t*-test with Welch’s correction where appropriate.

MSNs are a subpopulation of striatal neurons that are particularly vulnerable to neurodegeneration in HD. Therefore, we co-stained our neuronal cultures with the MSN marker DARPP32 to investigate whether RAN and RANGAP1 are mislocalized in this clinically relevant neuronal subpopulation (Figure 3A; Rosas et al., 2008; Johnson et al., 2020). Due to host species and antibody availability we were unable to co-stain DARPP32 and lamin-B1. From here on, we focused on the ESC-derived IsoHD lines (45Q and 81Q), and the IPSC-derived HD Family 75Q line as it closely matches the IsoHD 81Q length. Furthermore, mislocalization of RAN and RANGAP1 appeared consistent in neurons from 58 to 75Q. In HD Family line MSNs, we detected the same phenotype as in MAP2 + neurons, with a significantly lower RAN and RANGAP1 N/C in 75Q MSNs than in 22Q MSNs (Figures 3B,D). Notably, in IsoHD MSNs, the RAN N/C was significantly lower in both 45Q and 81Q MSNs than in 30Q MSNs, suggesting a more pronounced shift than in MAP2 + neurons (Figures 3C,E). Although no significant changes in the N/C ratio were observed in IsoHD MAP2 + neurons, 45Q and 81Q MSNs had a significantly lower RANGAP1 N/C ratio than their 30Q counterparts. Taken together, these findings suggest that IsoHD DARPP32 + /MAP2 + MSNs are more susceptible to nuclear pore deficits than DARPP32-/MAP2 + neurons and may contribute to the vulnerability of MSNs in HD.

### Human HD Pluripotent Stem Cell-Derived Astrocytes Do Not Appear to Exhibit Defects in Nuclear Pore Transport Protein Distribution

**FIGURE 4:**
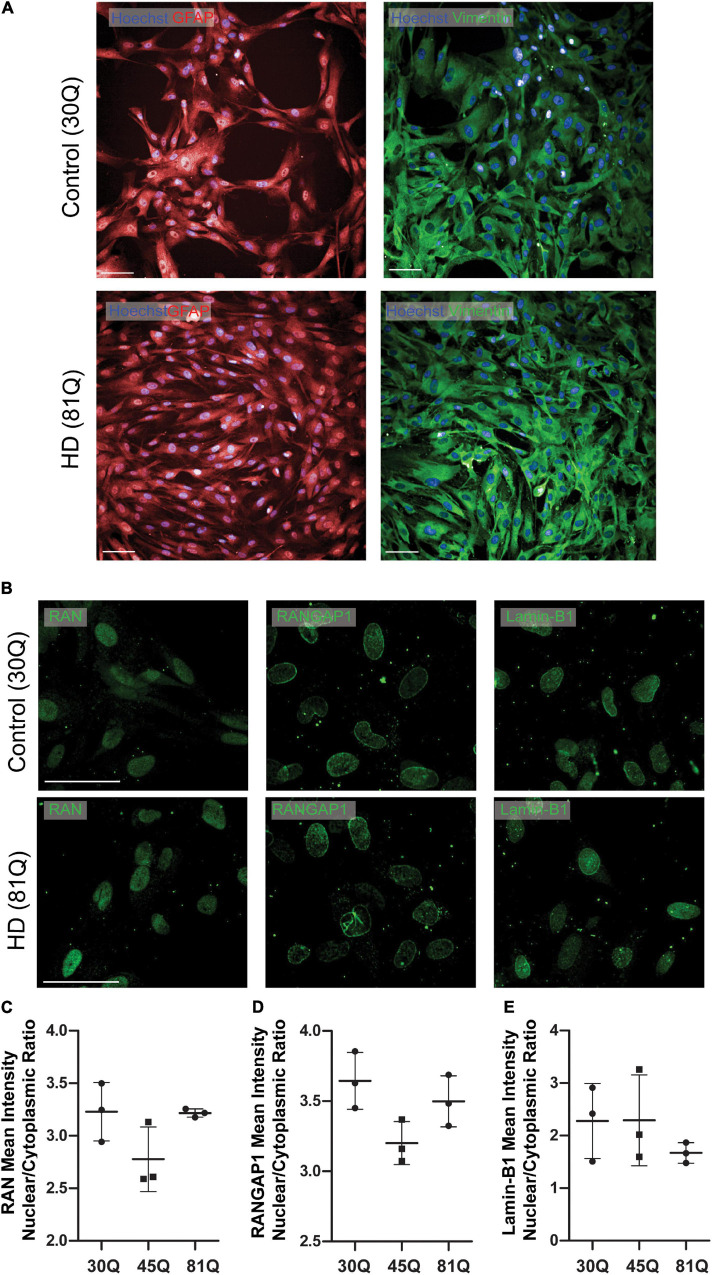
Human HD PSC- derived astrocytes do not exhibit nuclear pore defects. **(A)** HD ESCs (IsoHD 45Q, 81Q) and control ESCs (IsoHD 30Q) were differentiated into astrocytes and maintained for 3 weeks, after which they expressed astrocyte markers GFAP and VIMENTIN. Scale bars = 100 μM **(B)** Astrocytes were stained for the nuclear pore proteins; RAN, RANGAP1 and lamin-B1. **(C–E)** No significant differences were detected in the mean intensity nuclear/cytoplasmic ratio of RAN, RANGAP1 and lamin-B1. Data are presented as mean ± SEM of three differentiations of the IsoHD ESC line, analyzed by one-way ANOVA with Bonferroni correction.

As human HD PSC-derived striatal neurons exhibit robust nuclear pore protein mislocalization, we next wanted to examine whether these phenotypes extend to other neural cells derived from the same stem cell lines. Using the IsoHD series, both HD (45Q, 81Q) and unaffected PSCs (30Q) were successfully differentiated into astrocytes (Figure 4A and Supplementary Figure 8A) and stained for the nuclear pore protein markers RAN, RANGAP1 and lamin-B1 (Figure 4B and Supplementary Figure 8B), each of which were shown to be mislocalized in striatal neurons. Interestingly, IsoHD 45Q and 81Q astrocytes showed no evidence of a reduced mean intensity N/C ratio of these proteins (Figures 4C–E).

### Genes and Proteins Involved in Nuclear Pore Transport Are Dysregulated in Human HD Pluripotent Stem Cell-Derived Striatal Neurons

**FIGURE 5:**
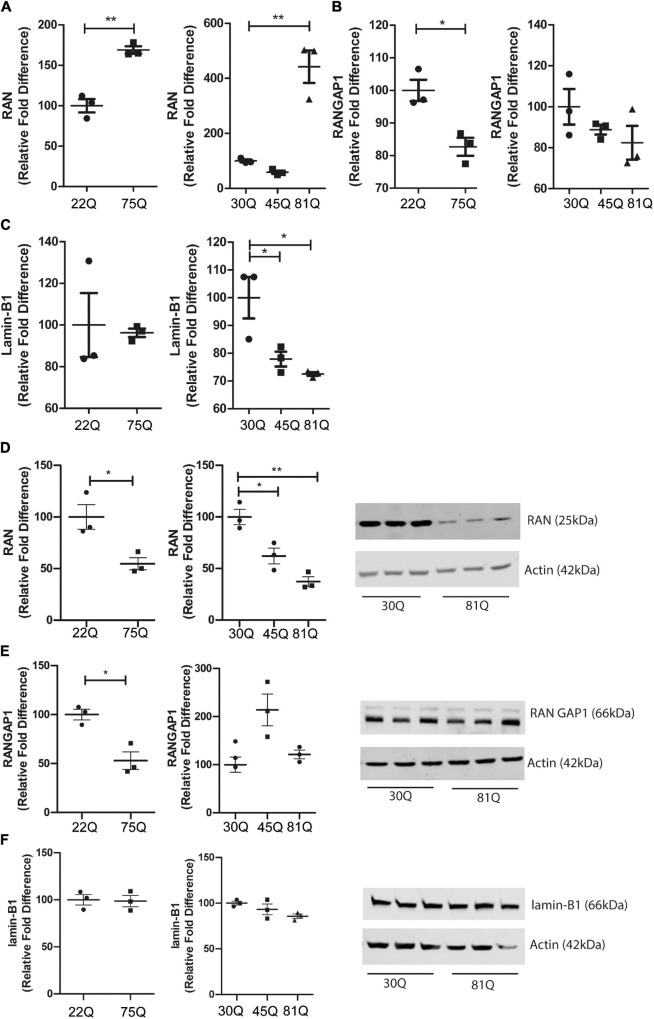
*RAN* gene and protein expression is altered in human HD PSC-derived striatal neurons. Expression of genes involved in nuclear cytoplasmic transport were assessed with qPCR in D37 striatal neurons and normalized to housekeeping genes EIF4A2, ATP5B and UBC. **(A)** Gene expression of *RAN* was significantly higher in HD Family line 75Q neurons and IsoHD 81Q neurons. **(B)** *RANGAP1* gene expression was significantly lower in HD Family line 75Q neurons than in 22Q neurons. A similar, non-significant trend was apparent in IsoHD 81Q neurons. **(C)** No significant differences were detected in gene expression of *LMNB1* in HD Family line neurons, however gene expression was significantly downregulated in IsoHD 45Q and 81Q neurons. Western blotting was used to assess protein expression and D37 neuronal samples were normalized to Actin (42 kDa). **(D)** Protein expression of RAN (25 kDa) was significantly lower in HD Family line 75Q neurons and IsoHD 45Q and 81Q neurons compared to their respective controls. **(E)** RANGAP1 (66 kDa) protein expression was significantly lower in HD Family line 75Q neurons than in 22Q neurons. No significant differences were detected in IsoHD striatal neurons. **(F)** No significant changes were apparent in lamin-B1 (66 kDa) protein expression in HD Family line neurons or IsoHD neurons. *: *p* < 0.05, **: *p* < 0.01. Data are represented as mean ± SEM of at least one differentiation of each clone of the HD Family iPSC lines, or three differentiations of each of the IsoHD ESC lines, subject to one-way ANOVA followed by Bonferroni *post hoc* test or unpaired *t*-test with Welsh’s correction where appropriate.

HTT has been shown to affect gene transcription (Kuhn et al., 2007; Benn et al., 2008). Therefore, we wanted to investigate whether genes involved in nuclear pore complex function are dysregulated. *RAN, RANGAP1 and Lamin-B1* gene expression was assessed in striatal neuron cultures at DD37 by RT-qPCR. *RAN* expression was significantly increased in both Family lines (75Q) and IsoHD lines (81Q) compared to their respective controls (Figure 5A). Expression of *RANGAP1* was significantly reduced in 75Q striatal cultures compared to control 22Q cultures and a similar trend, though not significant, was observed in IsoHD striatal cultures (Figure 5B). No significant changes in *Lamin-B1* were apparent in 75Q striatal neurons; however, *Lamin-B1* gene expression was significantly downregulated in IsoHD 81Q and 45Q cultures (Figure 5C).

We next quantified RAN, RANGAP1 and lamin-B1 protein levels in striatal neuron cultures by Western blotting. In contrast to the increased transcriptional expression of RAN, we found a significant reduction of total RAN protein in the 75Q cultures as compared to the 22Q controls, as well as significantly lower RAN protein expression in 45Q and 81Q compared to 30Q MSN controls (Figure 5D).

Protein levels of RANGAP1 were significantly lower in the HD Family 75Q neurons than in their 22Q control counterparts (Figure 5E) mirroring reduced gene expression. However, no significant differences in RANGAP1 protein were apparent in the isogenic line between 30Q and 45Q or 81Q striatal neuron protein samples (Figure 5E). We observed a band at 88kDa, which has been described as small ubiquitin-like modifier (SUMO)-2/3-conjugated RANGAP1 (Fujiwara et al., 2016), although the levels were low. This is in line with research showing that RANGAP1 is desumoylated as cells progress from cortical progenitors to terminally differentiated neuron (Fujiwara et al., 2016) and further supports that the majority of our striatal cultures are neuronal. In contrast, lamin-B1 protein expression was not affected in HD Family line neurons or IsoHD lines (Figure 5F). Although we saw a decrease in *Lamin-B1* gene expression in IsoHD neurons, this is not reflected in protein expression.

### Effect of Decreased Huntingtin Expression by Antisense Oligonucleotides on Nuclear Pore Transport Phenotypes

**FIGURE 6:**
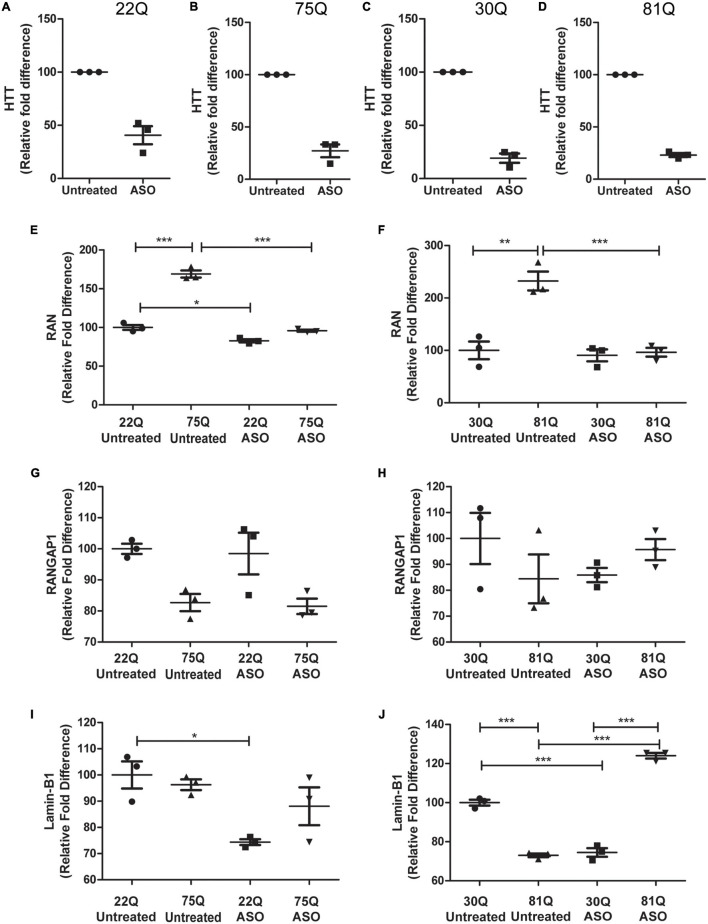
Total huntingtin (HTT) lowering with ASOs affects expression of genes encoding nucleocytoplasmic transport proteins. HD Family line 22Q and 75Q neuronal cultures (*n* = 3) and IsoHD 30Q and 81Q neuronal cultures (*n* = 3) were treated with ASOs from differentiation day 20 until day 37. **(A–D)** qPCR confirmed successful knockdown of *HTT*, normalized to housekeeping genes *EIF4A*, *UBC* and *ATP5B*. **(E,F)** Gene expression of *RAN* was significantly higher in HD neurons under basal conditions, but following ASO treatment no significant differences in RAN transcription were detected between control and HD neurons. **(G)** No significant changes were detected in *RANGAP1* gene expression in the HD Family line or **(H)** IsoHD neuronal cultures following ASO treatment. **(I)** ASO treatment significantly lowered *LMNB1* gene expression in control 22Q neurons, but not 75Q neuronal cultures. **(J)** In IsoHD neuronal cultures ASO treatment significantly increased *LMNB1* gene expression in 81Q neurons. *: *p* < 0.05, **: *p* < 0.01, ***: *p* < 0.001. Data are presented as mean ± SEM of at least one differentiation of each clone of the HD Family iPSC lines, or three differentiations of each of the IsoHD ESC lines, analyzed by one-way ANOVA with Bonferroni correction.

Lowering levels of insoluble HTT via small molecule approaches has been shown to reduce insoluble RANGAP1 aggregates (Grima et al., 2017). As aggregation of mHTT or nuclear pore proteins was not observed in our striatal neurons, we decided to take a different approach and lowered total *HTT* expression using antisense oligonucleotides (ASOs; Takeda Pharmaceuticals) delivered via nanoparticles in order to investigate whether this could normalize gene or protein expression, or restore the N/C ratios of RAN, RANGAP1 and lamin-B1 in human HD PSC-derived striatal neurons. ASO’s were delivered from DD20 until DD37, at which point samples were collected. The extent of *HTT* knockdown following treatment with anti-*HTT* ASOs of cells of the HD Family series was assessed by qPCR, showing a > 50% reduction in total *HTT* in 22Q samples and > 75% reduction in 75Q samples (Figures 6A,B), as well as in IsoHD 30Q and 81Q neuronal samples (Figures 6C,D). This was mirrored by reduced HTT protein expression in ASO treated 75Q and 81Q neuronal cultures (Supplementary Figure 9). Treatment with nanoparticles alone, lacking ASO did not result in *HTT* knockdown (Supplementary Figure 10). Importantly, neuronal viability was not affected by ASO treatment (Supplementary Figure 11).

As previously discussed, *RAN* expression was significantly higher under basal conditions in 75Q and 81Q neuronal cultures than their respective control. Following ASO treatment, *RAN* expression in HD Family 75Q and IsoHD 81Q neuronal cultures was reduced and comparable to that of their unaffected controls (Figures 6E,F), but no changes were observed at the protein level (Supplementary Figure 12). ASO treatment also reduced the *RAN* levels of 22Q neurons, although to a much smaller extent than in the HD lines. No significant changes were observed in *RANGAP1* gene expression following ASO treatment (Figures 6G,H), and gene expression appeared more variable in IsoHD neuronal cultures. Whilst reducing *HTT* expression did not significantly affect *Lamin-B1* expression in HD Family Line 75Q, which had comparable levels to untreated 22Q neuronal cultures, the *Lamin-B1* transcript was significantly upregulated in IsoHD 81Q neurons, which under basal conditions expressed significantly lower levels of *Lamin-B*1 than their respective control. In contrast, in both Family line 22Q and IsoHD 30Q unaffected neuronal cultures, ASO treatment appeared to downregulate *Lamin-B1* expression (Figures 6I,J). However this reduction in gene expression IsoHD 30Q, was not mirrored by a significant decrease in lamin-B1 protein, though it showed a trend in the same direction; no changes in lamin-B1 protein were observed in ASO-treated IsoHD 81Q neurons (Supplementary Figure 7).

**FIGURE 7:**
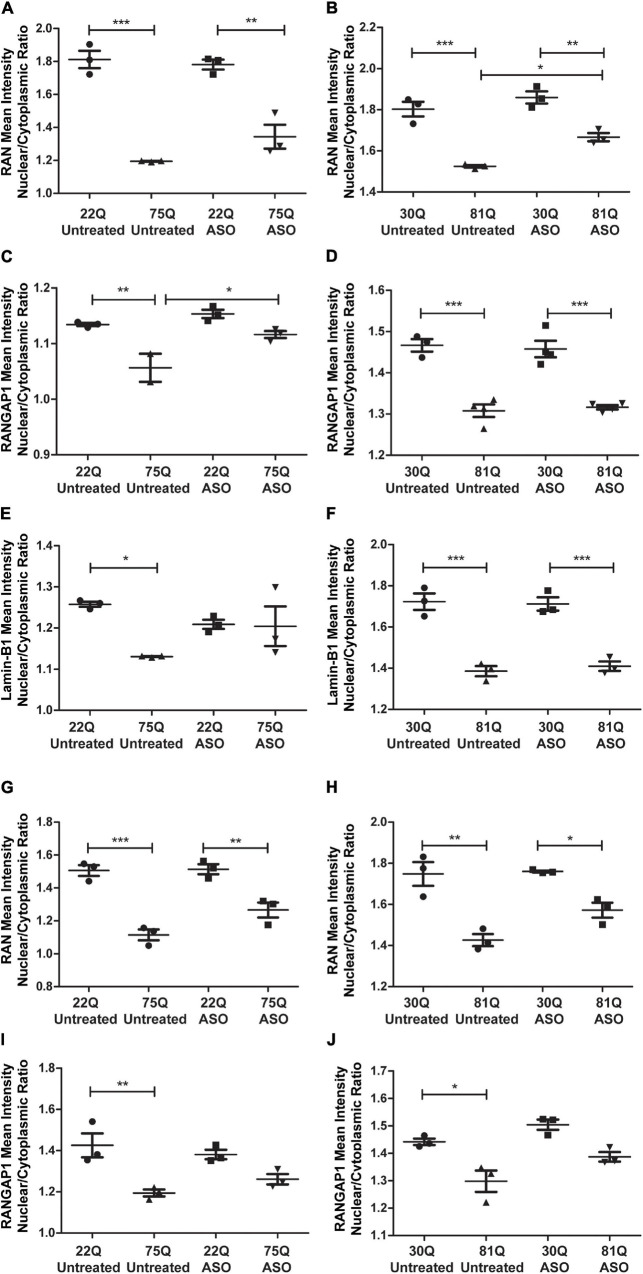
Total huntingtin (*HTT*) lowering with ASOs has a minor impact on nuclear pore protein localization. **(A,B)** In MAP2 + neurons the RAN nuclear/cytoplasmic (N/C) ratio remained significantly lower in ASO treated Family line and IsoHD neurons **(C)** Following ASO treatment no significant differences were detected in the RANGAP1 N/C between control and 75Q neurons, suggesting an increased N/C ratio **(D)**. In IsoHD cultures however, the RANGAP1 N/C remained significantly lower in IsoHD81Q neurons than their control. **(E)** Similarly, the lamin-B1 N/C ratio did not differ significantly between ASO treated Family line control and 75Q neurons **(F)** but remained significantly lower in ASO treated IsoHD81Q neurons than in IsoHD30Q neurons. **(G,H)** The RAN N/C remained significantly lower in ASO treated Family line 75Q and IsoHD81Q DARPP32 + MSNs than in their respective control **(I,J)**. No significant differences were detected in the RANGAP1 N/C ratio in HD Family line control 22Q and 75Q neurons, nor in the IsoHD 30Q or 81Q neurons following ASO treatment suggesting an increased N/C ratio. *: *p* < 0.05, **: *p* < 0.01, ***: *p* < 0.001. Data are presented as mean ± SEM of at least one differentiation of each clone of the HD Family iPSC lines, or three differentiations of each of the IsoHD ESC lines, analyzed by one-way ANOVA with Bonferroni correction.

Finally, we investigated whether ASO treatment corrected mislocalization of RAN, RANGAP1 and lamin-B1. In MAP2 + neurons, the RAN N/C ratio remained significantly lower in Family Line 75Q and IsoHD 81Q compared to their respective ASO treated control (Figures 7A,B) suggesting that lowering *HTT* is not sufficient to restore the RAN N/C ratio. However, in ASO treated 81Q striatal cultures, the RAN N/C was significantly higher than in untreated cultures. ASO treatment significantly increased the RANGAP1 N/C ratio in 75Q neurons compared to untreated 75Q neurons (Figure 7C). Similarly, a trend toward an increased lamin-B1 N/C ratio was observed in these cultures (Figure 7E). No significant differences were detected in the RANGAP1 and lamin-B1 N/C ratio between Family line 22Q control and 75Q HD neurons following ASO treatment, whereas in untreated HD cultures the N/C ratio was significantly lower than in 22Q neurons (Figures 7C,E). However in IsoHD neuronal cultures no significant changes were detected between untreated and ASO treated 81Q neurons, and the RANGAP1 and lamin-B1 N/C ratio remained significantly lower in 81Q neurons than in 30Q neurons following ASO treatment (Figures 7D,F). In both Family line and IsoHD DARPP32 + /MAP2 + neurons, a slight but non-significant increase was observed in the RAN N/C ratio of ASO treated 75Q and 81Q neurons compared to untreated 75Q and 81Q neurons (Figures 7G,H). Furthermore the RAN N/C ratio remained significantly lower than that of their respective control line (22Q, 30Q) (Figures 7G,H). We detected a similar small, but non-significant increase in the RANGAP1 N/C ratio of ASO treated 75Q and 81Q DARP32 + /MAP2 + neurons. Notably no significant differences were detected in the RANGAP1 N/C ratio between Family line 75Q neurons and 22Q DARPP32 + /MAP2 + neurons, nor IsoHD81Q and 30Q DARPP32 + /MAP2 + neurons indicating that that ASO treatment was able to reduce RANGAP1 mislocalization to a limited extend (Figures 7I,J). ASO treatment did not impact neuronal morphology in HD or control neurons, suggesting that changes in localization are not due to morphological changes within neurons (Supplementary Figure 13). Here we show that total HTT lowering induced changes in nuclear pore transport gene expression, as well as subtle changes in RANGAP1 and RAN localization.

## Discussion

Nuclear pore deficits and nucleocytoplasmic transport deficits present an emerging phenotype in HD and other neurodegenerative diseases (Kim and Taylor, 2017; Sakuma and D’Angelo, 2017; Lester and Parker, 2018), having been extensively characterized in post-mortem human brains, mouse models and primary cell cultures (Gasset-Rosa et al., 2017; Grima et al., 2017). To date, this has been described only to a limited extent in human PSC-derived cell cultures (Gasset-Rosa et al., 2017; Grima et al., 2017). In this study we sought to assess whether NPC dysfunction is present in PSC-derived striatal neuron cultures that contain a proportion of the DARPP32 + medium spiny neurons that are amongst the most vulnerable cell populations in HD. We used a series of highly genetically related iPSC lines (with 22, 58, 69 and 75 polyQ repeat lengths) and isogenic ESC lines (with 30, 45 and 81 polyQ repeat lengths) (Ooi et al., 2019). Variability between lines is one of the major limitations in working with PSC derived cultures (Kyttälä et al., 2016; Carcamo-Orive et al., 2017) and highlights the need for using more than one cell line with isogenic controls to obtain robust, replicable results. Although our patient derived lines are highly genetically related, we anticipated more variability in the HD Family line than in the ESC-derived isogenic lines. Despite this, we observed reproducible phenotypes across both PSC-derived neuronal cultures.

### High Content Imaging Can Detect Nucleocytoplasmic Transport Phenotypes in Striatal Neurons

We decided to use high content imaging as an unbiased, robust and high-powered means of addressing confounding issues of genetic and technical variability, whilst determining the presence or otherwise of typical pathological hallmarks and phenotypes in cultures of the human striatal neurons that are particularly affected in Huntington’s disease. An additional benefit is the ability of high content imaging to analyze large numbers of samples in a relatively short timeframe. This means that, unlike common fluorescence microscopy techniques, observations made in the undertaking of basic scientific research can be scaled up for use in high throughput drug discovery with relative ease (Ketteler and Kriston-Vizi, 2016; Little et al., 2018, 2019; Sherman and Bang, 2018).

Mislocalization of RAN and RANGAP1, as well as aggregation of the latter with mHTT have been previously studied thoroughly in mouse models and human post mortem brains, but only to a limited extent in IPSC-derived neurons (Grima et al., 2017). Given the complicated nature of reproducibility in IPSC derived models of disease (Volpato and Webber, 2020), it was important to validate nuclear pore phenotypes on a larger scale, increasing the number of MAP2 + neurons assessed by at least tenfold. Indeed, the imaging approach used here identified mislocalization of RAN and RANGAP1 across HD lines, thus establishing that high content imagining is a suitable method for detecting nuclear pore deficits as well as confirming that disruption of nuclear pore transport proteins is a consistent HD phenotype in a highly powered study.

Aberrant nuclear architecture has been reported in several neurodegenerative disease models and our analysis of human neurones derived from different HD lines further supports the view that this is also a feature of HD (Edens et al., 2013; Frost et al., 2016; Gasset-Rosa et al., 2017; Alcalá-Vida et al., 2021). Lamin-B1 is a nuclear envelope protein that is highly expressed in neurons and provides structural support to the nucleus (Stuurman et al., 1998; Takamori et al., 2018). It previously been used as a test of nuclear integrity in HD studies, as atypical nuclear structure presents a hallmark of aging in cells (Scaffidi and Misteli, 2006; López-Otín et al., 2013) that has been proposed to be accelerated in HD (Gasset-Rosa et al., 2017). Our high content imaging analysis detected a disruption of the nuclear cytoplasmic ratio of lamin-B1, in MAP2 + neurons indicating that NPC defects extend beyond active transport and extend to the nuclear envelope.

### Nuclear Pore Transport Defects Are Present in Medium Spiny Neurons

Striatal MSNs are particularly vulnerable at early stages of HD progression (Rosas et al., 2008; Johnson et al., 2020), but whether this neuronal subpopulation presents abnormal nuclear pore phenotypes had not been investigated. High content imaging revealed that mislocalization of RAN and RANGAP1 is present in MSNs. Crucially, it revealed an abnormal phenotype in the adult onset IsoHD 45Q MSNs, which was not apparent when the whole IsoHD 45Q neuronal population (MAP2 + neurons) was analyzed. These findings may indicate that MSNs are particularly vulnerable to disruptions in nuclear pore transport and mislocalization of RAN and RANGAP1. It is also of note that nuclear pore transport defects were not apparent in HD astrocytes, demonstrating specificity of nuclear pore defects to selected neural cell types. Research has suggested that unique nucleoporin combinations can generate NPCs with specialized functions, which may be region and also cell type dependent (Raices and D’Angelo, 2012). It is possible that these cell type specific NPCs result in HD astrocytes being more resistant to nuclear pore dysfunction whereas MSNs appear more susceptible (Garcia-Segura et al., 1989).

Given the close interaction between RAN and RANGAP1, it is possible that disruption of one might have a knock-on effect on the other. Nucleocytoplasmic changes of RAN and other nuclear pore proteins has also been linked to an early phenotype of cellular stress and apoptosis (Ferrando-May et al., 2001; Ferrando-May, 2005; Moss et al., 2009; Bano et al., 2010). Increased relocation of RAN to the cytoplasm has been observed following treatment with stressors such as hydrogen peroxide and is associated with reduced import activity (Yasuda et al., 2006). It is possible that nuclear pore protein mislocalization is an early HD phenotype and an indicator of cellular stress in MSNs.

HD is associated with mHTT-positive inclusions that are found in the nuclei and neurites of susceptible neurons (DiFiglia et al., 2016). Whilst inclusion frequency and rate of formation are polyglutamine-length dependent and inclusion size increases with disease duration (Scherzinger et al., 1999), the definitive effects of these inclusions have not yet been established and do not appear to explain the particular vulnerability of MSNs (Ehrlich, 2012). Aberrant co-localization of nuclear pore proteins with mHTT has been reported in mouse models, human post-mortem brains and murine primary cultures (Gasset-Rosa et al., 2017; Grima et al., 2017). In contrast to post-mortem HD brain, HTT inclusions have not been consistently observed in human HD PSC-derived models. Our results showing no differences in aggregate markers, nor in aggregation of RANGAP1 or other nuclear pore proteins that may associate with mHTT in HD neurons are consistent with this and indicate that occurrence of nuclear pore deficits precede, rather than being a consequence, of mHTT aggregation. This further supports the view that nuclear pore protein abnormalities described in our study likely represents an early phenotype of relatively young HD neurones that precedes the aggregation of such proteins.

### Nuclear Pore Transport Components Are Disrupted on a Transcriptional and Protein Level in Huntington’s Disease

To date no studies have examined whether nuclear pore transport components are disrupted on a transcriptional or protein level in HD except in the context of aggregation (Grima et al., 2017). We observed significantly elevated transcription of *RAN*, in contrast to reduced protein expression in HD neurons. Whereas the activity of the Ran-RANBP1-RANGAP complex has been investigated (Seewald et al., 2003), little is known about RAN turnover regulation. Nonetheless, it is tempting to speculate that significant reduction of RAN protein in HD neurones, possibly due to increased catabolism, might stimulate RAN transcription via feed-back mechanisms. In contrast, reduced transcription of *RANGAP1* (Family line) and *Lamin-B1* (IsoHD line) was mirrored by reduced protein expression. Reduced RANGAP1 expression was observed in HD mice at late disease stages (Grima et al., 2017), whereas we found reduced RAN and RANGAP1 protein levels in our “young” PSC-derived neurones, suggesting that nuclear pore protein reduction may be an earlier phenotype than previously indicated through its links with aggregation. Interestingly increased lamin-B1 protein expression has been described in an HD mouse model, and further overexpression of lamin-B1 resulted in morphological changes in neurons (Alcalá-Vida et al., 2021). We did not observe increased lamin-B1 expression, possibly due to lower polyQ lengths of our neurons compared to the mouse model. It has yet to be established whether changes in nuclear pore mRNA and protein expression are directly linked to the mislocalization of RAN and RANGAP1 that we have observed. Indeed, despite changes in *RAN* and *Lamin-B1* transcription following HTT knockdown, no restoration of protein levels was apparent nor a correction in their localization.

### Lowering Huntingtin Is Not Sufficient to Restore Mislocalisation of Nuclear Pore Transport Proteins in Huntington’s Disease Neurons

A previous study has shown that lowering insoluble mHTT aggregates leads in turn to reduced RANGAP1 aggregation and that overexpression of RAN and RANGAP1 increased neuronal viability in primary cultures (Grima et al., 2017). However, it did not show whether this also reduced the nuclear/cytoplasmic ratio imbalance and restored localization of nuclear pore proteins.

Here we have down-regulated HTT levels using antisense oligonucleotides and shown that despite a robust HTT knockdown was achieved in both control and HD neurons, only observed subtle changes in the RAN N/C ratio either in MAP2 or DARPP32 -positive neurons, suggesting that some active transport deficits remain. We did not detect significant differences in the RANGAP1 and lamin-B1 N/C ratio following ASO treatment in Family line 75Q vs control MAP2 + neurons, as well as an significant increase in the RANGAP1 N/C ratio in 75Q neurons indicating some restoration of the RANGAP1 N/C ratio. This did not extend to the IsoHD line, raising the possibility that other genetic modifiers may play a role. In both Family line and IsoHD DARPP32 + /MAP2 + neurons, the RANGAP1 N/C ratio was subtly increased between untreated and ASO treated HD neurons and was not detectably different between control and HD MSNs. There are several possibilities why lowering *HTT* may only have subtle effects on nuclear pore protein localization as well as being restricted to specific proteins such as RANGAP1. Given the slow turnover of some nuclear pore complex proteins (Daigle et al., 2001; D’Angelo et al., 2009; Sakuma and D’Angelo, 2017), a longer ASO treatment in long-term neuronal cultures may be necessary to induce more noticeable changes. It is also possible that the aberrant phenotypes observed in HD neuronal cultures are the result of a toxic gain of function of mHTT. As ASOs lowered total HTT levels, mHTT levels may have not been lowered sufficiently or alternatively normal HTT protein levels need to be increased. If HTT directly interacts with nuclear pore proteins or factors downstream of HTT contribute to the nuclear pore transport phenotypes we observed, restoring normal HTT levels may be necessary to correct nuclear pore protein localization. Indeed, it has been suggested that HTT may function as a nuclear transport factor and promotes NF-kB transport to the nucleus, but this function is impaired when a polyglutamine expansion is present (Marcora and Kennedy, 2010). In this case CRISPR/Cas would be a more suitable option than the use of ASOs (Nakamori et al., 2020). It is also possible that nuclear pore deficits are a common phenotype in degenerating neurons that are accelerated by the presence of mHTT. Nuclear pore deficits have been proposed to underlie normal cellular aging (López-Otín et al., 2013). In healthy aged rats, composition of NPCs were altered and undergo oxidative damage (Patel and Chu, 2011). Oxidative stress has been linked to increased leakiness of the nuclear envelope, as well as to a collapse of the RAN -GTP gradient. Several HD models have suggested that mHTT accelerates these phenotypes (Gasset-Rosa et al., 2017; Veldman and Yang, 2017). mHTT may worsen nuclear pore complex phenotypes common to neurodegeneration and lead to the pronounced defects observed in our striatal cultures, which cannot completely be alleviated by a reduction in total HTT.

We have shown that high content imaging of human HD PSC-derived neuronal cultures can reduce variability due to small scale PSC studies, and revealed disruption of active nuclear transport-associated proteins in HD neurons and MSNs that was robustly replicated in several PSC lines. Additionally, we have also demonstrated the use of this high content screening platform for potential therapeutic drug treatment in neuronal cultures and, though ASO treatment was not found to be effective in completely reversing nuclear pore phenotypes, this system presents a useful platform for screening further treatments for nuclear pore transport phenotypes.

## Data Availability Statement

The raw data supporting the conclusions of this article will be made available by the authors, without undue reservation.

## Author Contributions

JL, AW-K, YU, AK, RA, and ST designed the research. JL, AW-K, AA, CC, RG, SF, JP, and RA performed the research. JL, AW-K, and RA analyzed the data. JL wrote the manuscript. PF input on the scientific background of the manuscript and was involved in the compilation of the manuscript. All authors commented on the final version of the manuscript.

## Conflict of Interest

YU and AK were employed by the company Takeda Pharmaceutical Company Limited. This study received funding from Takeda Pharmaceuticals Ltd. The funder was involved in the study design. This study received funding from Cerevance, Inc. Cerevance, Inc., was not involved in the study design, collection, analysis, interpretation of data, the writing of this article, or the decision to submit it for publication. The remaining authors declare that the research was conducted in the absence of any commercial or financial relationships that could be construed as a potential conflict of interest.

## Publisher’s Note

All claims expressed in this article are solely those of the authors and do not necessarily represent those of their affiliated organizations, or those of the publisher, the editors and the reviewers. Any product that may be evaluated in this article, or claim that may be made by its manufacturer, is not guaranteed or endorsed by the publisher.

## Supplementary Material

Supplementary Figure 1 Control and Huntington’s disease PSC-derived medium spiny neuron cultures contain similar percentages of neural progenitor and proliferating cells. **(A)** Neuronal cultures were stained with proliferation marker Ki67 and counterstained with Hoechst. The percentages of proliferating cells were similar in HD Family and IsoHD lines and no significant changes were detected between control and HD cultures. **(B)** Staining for neural progenitor marker Nestin, which accounted for 5–7% of cells, showed no significant differences between lines or control and HD cultures.

Supplementary Figure 2 Huntington’s disease PSC-derived medium spiny neuron cultures show no evidence of HTT inclusion formation. The numbers of **(A,B)** S830 + and **(C,D)** EM48 + spots in the nuclei of HD Family and IsoHD lines was assessed. Spots were detectable in all lines, and data were normalized by subtracting the mean control value from each data point. There was no significant HTT polyglutamine-length differences in spot frequency or number of nuclei containing a threshold number of spots that might have been indicative of the formation of aggregated HTT. Data are represented as mean ± SEM Huntington’s disease human pluripotent stem cell-derived medium spiny neurons of at least one differentiation of each clone of the HD Family iPSC lines, or three differentiations of each of the IsoHD ESC lines, subject to one-way ANOVA followed by Dunnett *post hoc* test.

Supplementary Figure 3 Immunostaining of nuclear pore proteins Ran, RANGAP1 and nuclear envelope marker lamin-B1 in HD PSC-derived neuronal striatal cultures. Neuronal cultures were stained for lamin-B1, RAN and RANGAP1 and were counterstained with Hoechst. In HD neurons, lamin-B1, RAN and RANGAP1 are detected much more widely in soma and processes indicated by the arrows and outline of the nuclear area, compared to control cultures where expression is highly localized to the nucleus. Scale bar = 20 μM.

Supplementary Figure 4 Mean staining Intensities of nuclear pore proteins Ran, RANGAP1 and nuclear envelope marker lamin-B1 in HD PSC-derived neuronal striatal cultures. **(A)** No significant differences were detected in mean intensity of RAN in the nucleus of Family line and IsoHD nuclei. **(B)** Mean Intensity of RAN appears higher in the cytoplasm of Family line 58Q, 69Q, 75Q neurons and IsoHD 81 neurons. **(C)** No significant differences were detected in mean intensity of RANGAP1 in the nucleus of Family line and IsoHD nuclei. **(D)** Mean Intensity of RANGAP1 appears higher in the cytoplasm of Family line 58Q, 69Q, 75Q neurons and IsoHD 81 neurons. **(E)** No significant differences were detected in mean intensity of lamin-B1 in the nucleus of Family line and IsoHD nuclei. **(F)** Mean intensity of lamin-B1 appeared higher in the Family line 75Q neurons and was significantly higher in Family line 58Q,69Q, and IsoHD 81 neurons. *: *p* < 0.05, **: *p* < 0.01. Data are presented as mean ± SEM of at least one differentiation of each clone of the HD Family iPSC lines, or three differentiations of each of the IsoHD ESC lines, analyzed by one-way ANOVA with Bonferroni correction.

Supplementary Figure 5 Huntington’s disease PSC-derived medium spiny neuron cultures exhibit no overt morphological abnormalities. **(A)** Nuclear size was measured in neurons stained for MAP2 and Hoechst and no significant changes were detected between control and HD neurons in Family or IsoHD lines. **(B)** Cytoplasmic size of MAP2 + neurons also showed no significant changes between control and HD. **(C)** No significant changes were detected in the cytoplasm size of DARPP32 + /MAP2 + neurons.

Supplementary Figure 6 Huntington’s disease PSC-derived medium spiny neuron cultures show no evidence of RAN aggregation. MAP2 + neurons stained for RAN exhibited RAN positive puncta, however the percentage of neurons with more than one RAN positive puncta in the nucleus **(A)** or cytoplasm **(B)** did not differ significantly between control and HD neurons in either Family HD or IsoHD lines.

Supplementary Figure 7 Nucleoporins are mislocated in isogenic, but not Family line HD PSC-derived striatal neuronal cultures. **(A)** Neuronal cultures were stained with MAP2 and nucleoporins (NUP) 62 and **(B)** NUP88. Scale bar = 20 μM. **(C)** No significant differences were detected in the nuclear/cytoplasmic (N/C) ratio of NUP62 for the HD Family lines (58Q, 69Q, and 75Q). The N/C ratio of NUP62 was significantly lower in IsoHD 81Q neurons. **(D)** No significant differences were detected in the N/C ratio of NUP88 for the HD Family lines (58Q, 69Q, and 75Q). IsoHD 81Q neurons exhibited a significantly lower N/C ratio for NUP88, but not IsoHD 45Q neurons. *: *p* < 0.05, **: *p* < 0.01, ****: *p* < 0.0001. Data are presented as mean ± SEM of at least one differentiation of each clone of the HD Family iPSC lines, or three differentiations of each of the IsoHD ESC lines, analyzed by one-way ANOVA with Bonferroni correction.

Supplementary Figure 8 Immunostaining Huntington’s disease PSC-derived astrocytes (45Q) for astrocyte markers and nuclear pore proteins. **(A)** IsoHD 45Q astrocytes stained positively for intermediate filament markers GFAP and Vimentin, confirming astrocyte identity. **(B)** IsoHD 45Q astrocytes were stained for RAN, RANGAP1 and lamin-B1 to investigate nuclear pore transport deficits.

Supplementary Figure 9 Antisense oligonucleotide treatment successfully lowers huntingtin (HTT) protein levels in HD neurons. **(A)** Western blot of HTT and loading control Actin in untreated and ASO treated HD Family 75Q and IsoHD 81Q neuronal cultures at day 37. ASO treatment reduced full length HTT (350 kDa) as well as lower molecular weight fractions. **(B)** Full length HTT expression was normalized to Actin and measured.

Supplementary Figure 10 Treatment with Nanoparticles (vehicle) has no effect on huntingtin (*HTT*) transcript levels. QPCR confirmed that treatment with nanoparticles only did not lower *HTT* in control (22Q) or HD (75Q) neurons. Values were normalized to housekeeping genes UBC, ATP5B and EIF4A.

Supplementary Figure 11 Treatment with antisense oligonucleotides (ASO) to huntingtin (HTT) does not affect viability of neuronal cultures. The percentage of viable nuclei was determined by quantifying pyknotic or dysmorphic nuclei as an inverse proportion of the total number of cells. ASO treated cultures were normalized to their untreated counterparts and we did not detect any adverse effects of ASO treatment on neuronal viability. Data are presented as mean ± SEM of three clones of 22Q and 75Q, analyzed by one-way ANOVA with Bonferroni correction.

Supplementary Figure 12 Treatment with antisense oligonucleotides (ASO) to huntingtin (HTT) does not affect lamin-B1 and RAN protein expression in HD neurons. **(A)** Western blot of lamin-B1, RAN and loading control Actin in untreated and ASO treated IsoHD 30Q and 81Q neuronal cultures at day 37. **(B)** ASO treatment had no significant impact on lamin-B1 protein levels in IsoHD 30Q and 81Q neurons **(C,D)**. No significant differences were detected in RAN protein expression in HD Family line control 22Q and 75Q cultures or IsoHD control 30Q and 81Q neuronal cultures.

Supplementary Figure 13 Treatment with antisense oligonucleotides (ASO) to huntingtin (HTT) does not affect neuronal morphology. **(A)** Nuclear size and **(B)** cytoplasmic size of MAP2 + neurons, as well **(C)** cytoplasmic size of DARP32 + /MAP2 + neurons were not altered by ASO treatment.
