# Supplementary figures and images for: Mislocalization of Nucleocytoplasmic Transport Proteins in Human Huntington’s Disease PSC-Derived Striatal Neurons
Source: Front Cell Neurosci. 2021 Sep 29;15:742763. doi: 10.3389/fncel.2021.742763 (PMC8519404; doi:10.3389/fncel.2021.742763)

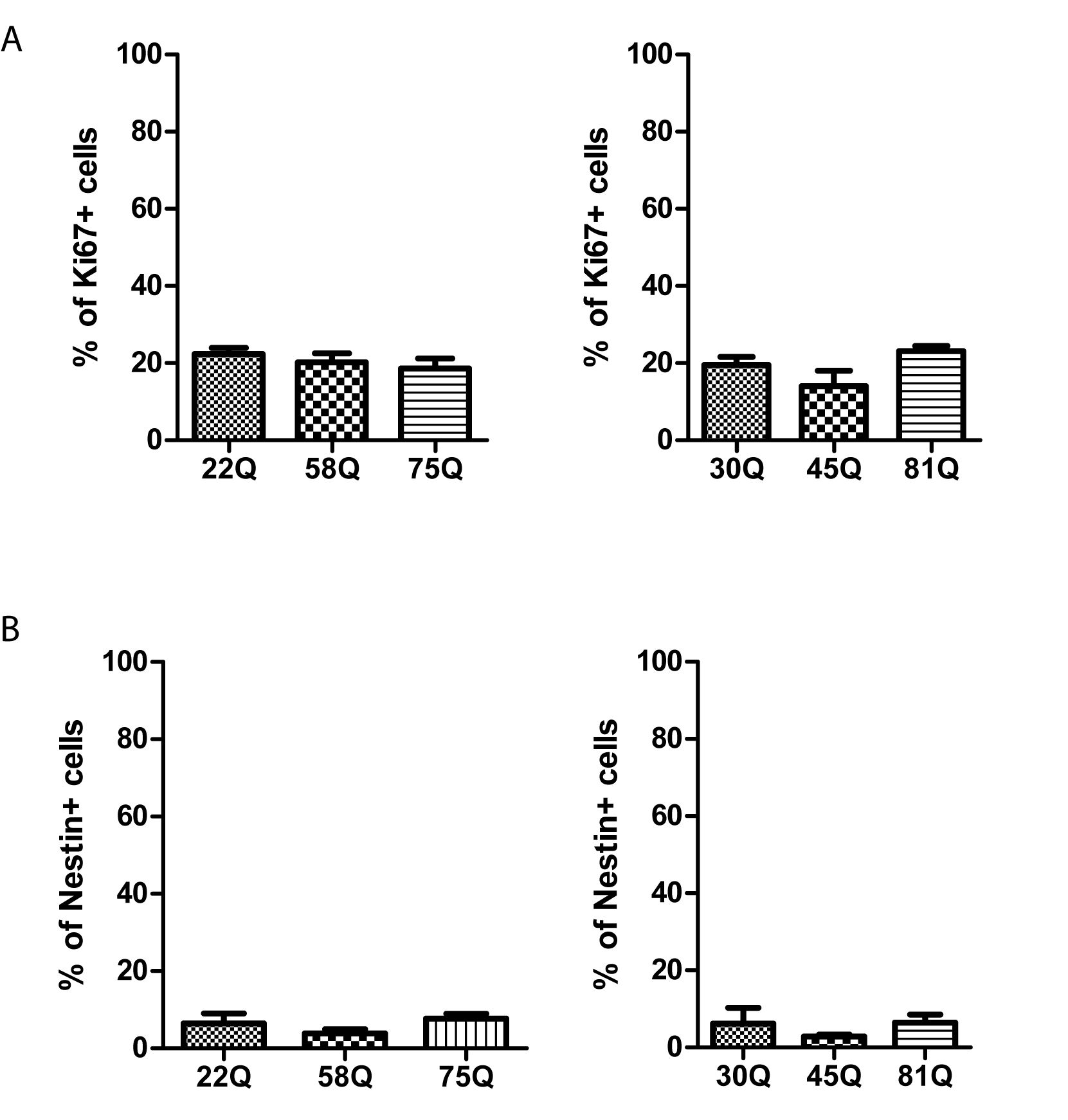

Supplement: Supplementary Figure 1 — Control and Huntington’s disease PSC-derived medium spiny neuron cultures contain similar percentages of neural progenitor and proliferating cells. (A) Neuronal cultures were stained with proliferation marker Ki67 and counterstained with Hoechst. The percentages of proliferating cells were similar in HD Family and IsoHD lines and no significant changes were detected between control and HD cultures. (B) Staining for neural progenitor marker Nestin, which accounted for 5–7% of cells, showed no significant differences between lines or control and HD cultures. [file Image_1.TIF]

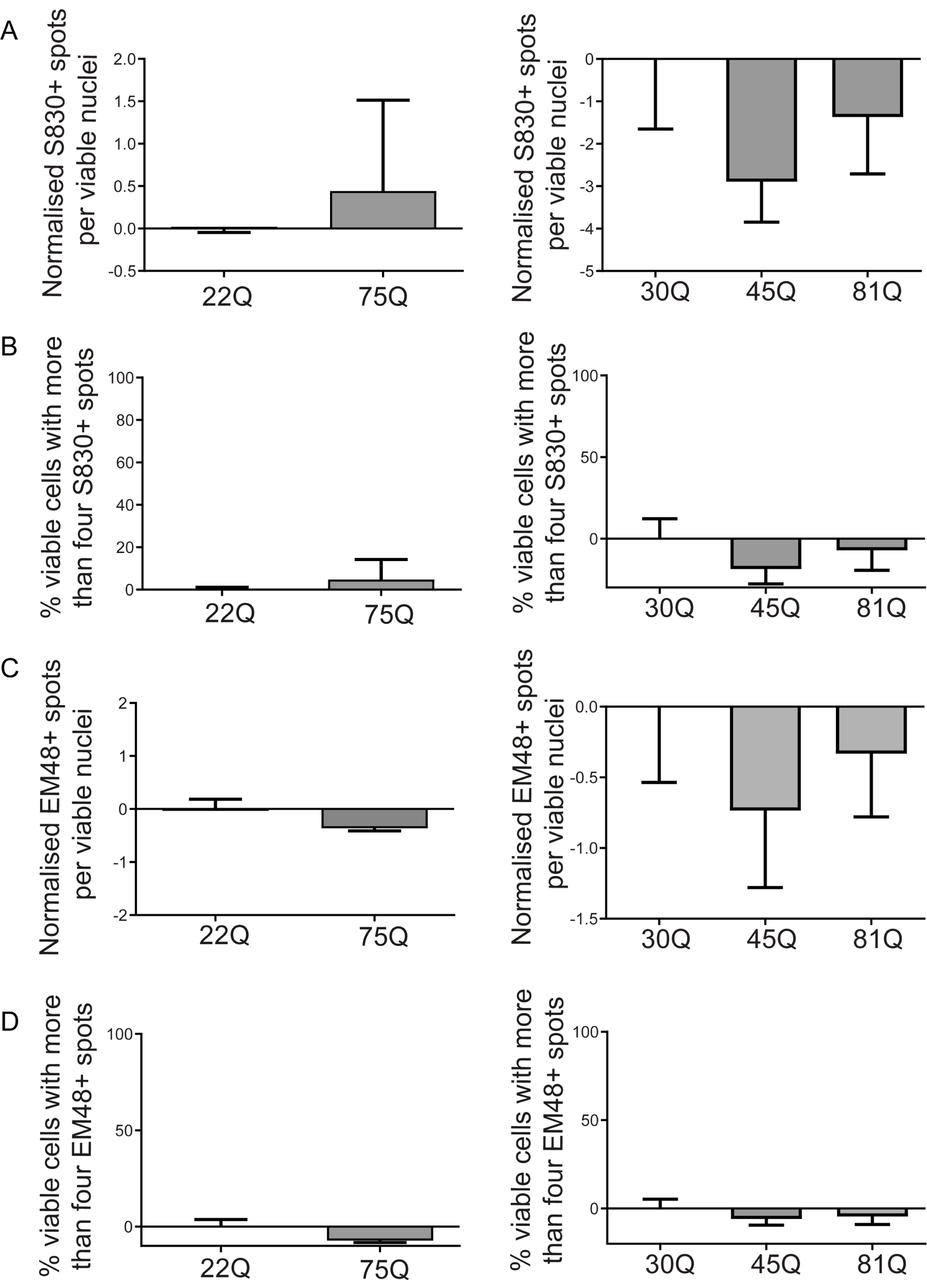

Supplement: Supplementary Figure 2 — Huntington’s disease PSC-derived medium spiny neuron cultures show no evidence of HTT inclusion formation. The numbers of (A,B) S830 + and (C,D) EM48 + spots in the nuclei of HD Family and IsoHD lines was assessed. Spots were detectable in all lines, and data were normalized by subtracting the mean control value from each data point. There was no significant HTT polyglutamine-length differences in spot frequency or number of nuclei containing a threshold number of spots that might have been indicative of the formation of aggregated HTT. Data are represented as mean ± SEM Huntington’s disease human pluripotent stem cell-derived medium spiny neurons of at least one differentiation of each clone of the HD Family iPSC lines, or three differentiations of each of the IsoHD ESC lines, subject to one-way ANOVA followed by Dunnett post hoc test. [file Image_2.TIF]

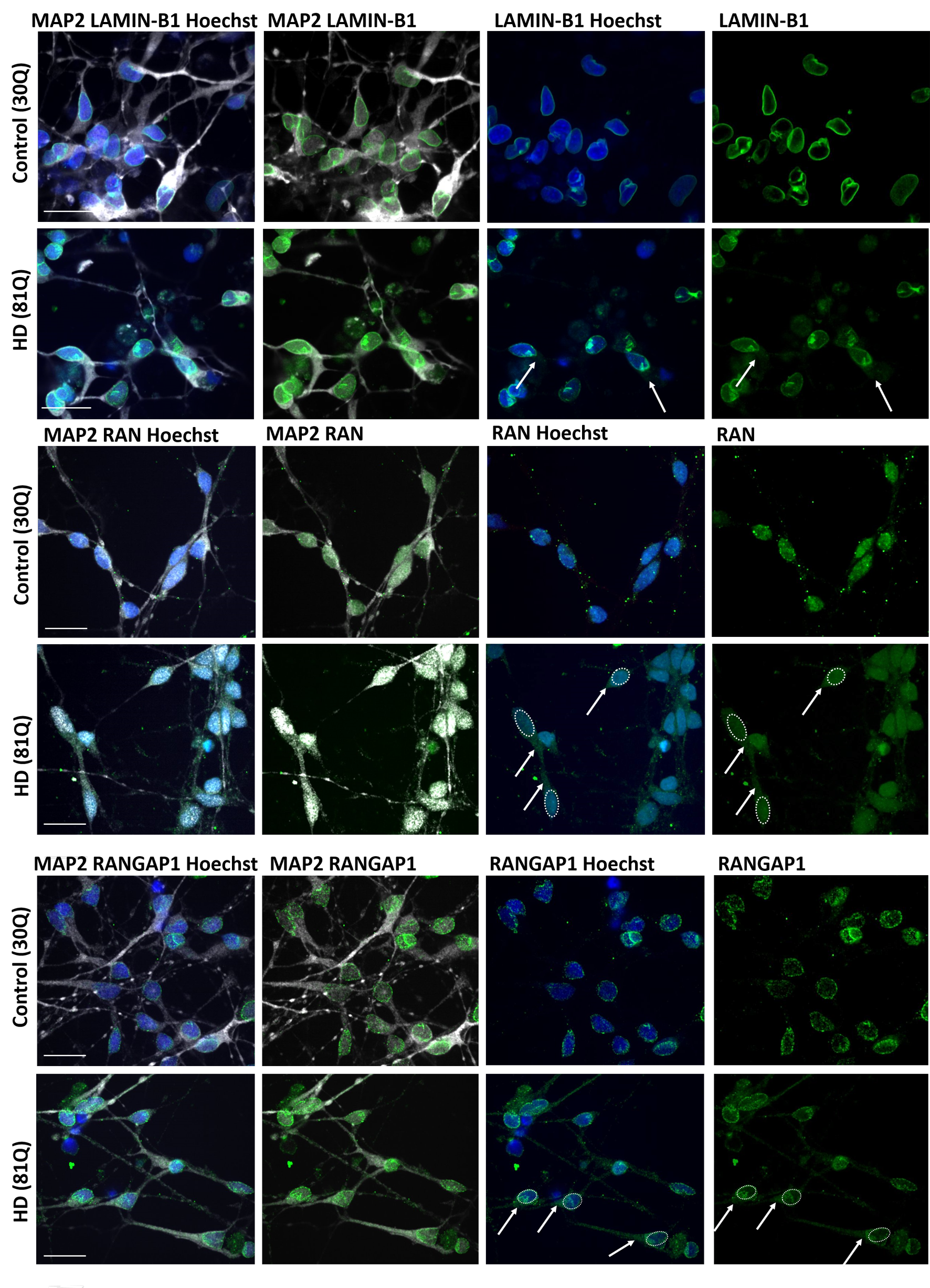

Supplement: Supplementary Figure 3 — Immunostaining of nuclear pore proteins Ran, RANGAP1 and nuclear envelope marker lamin-B1 in HD PSC-derived neuronal striatal cultures. Neuronal cultures were stained for lamin-B1, RAN and RANGAP1 and were counterstained with Hoechst. In HD neurons, lamin-B1, RAN and RANGAP1 are detected much more widely in soma and processes indicated by the arrows and outline of the nuclear area, compared to control cultures where expression is highly localized to the nucleus. Scale bar = 20 μM. [file Image_3.TIF]

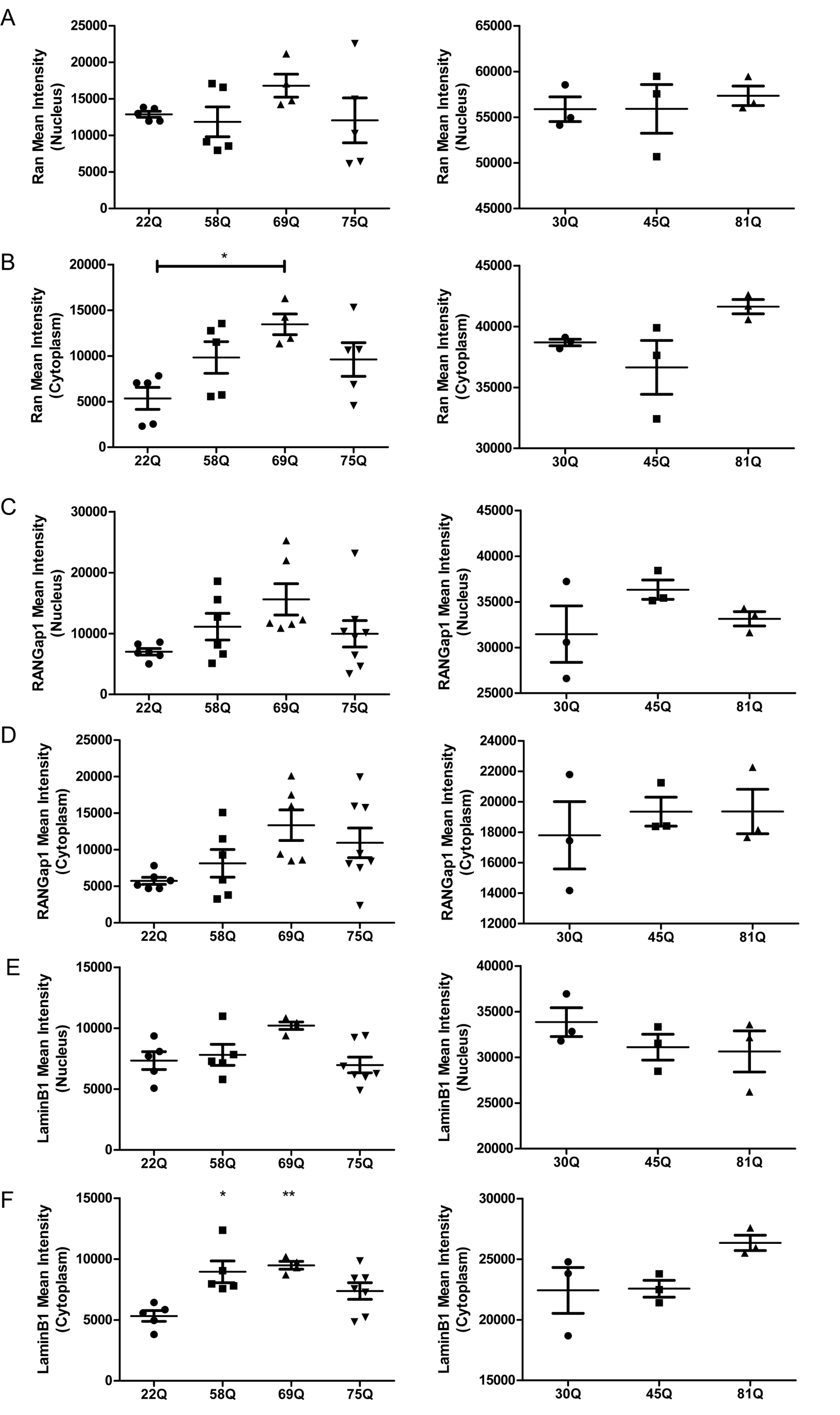

Supplement: Supplementary Figure 4 — Mean staining Intensities of nuclear pore proteins Ran, RANGAP1 and nuclear envelope marker lamin-B1 in HD PSC-derived neuronal striatal cultures. (A) No significant differences were detected in mean intensity of RAN in the nucleus of Family line and IsoHD nuclei. (B) Mean Intensity of RAN appears higher in the cytoplasm of Family line 58Q, 69Q, 75Q neurons and IsoHD 81 neurons. (C) No significant differences were detected in mean intensity of RANGAP1 in the nucleus of Family line and IsoHD nuclei. (D) Mean Intensity of RANGAP1 appears higher in the cytoplasm of Family line 58Q, 69Q, 75Q neurons and IsoHD 81 neurons. (E) No significant differences were detected in mean intensity of lamin-B1 in the nucleus of Family line and IsoHD nuclei. (F) Mean intensity of lamin-B1 appeared higher in the Family line 75Q neurons and was significantly higher in Family line 58Q,69Q, and IsoHD 81 neurons. *: p < 0.05, **: p < 0.01. Data are presented as mean ± SEM of at least one differentiation of each clone of the HD Family iPSC lines, or three differentiations of each of the IsoHD ESC lines, analyzed by one-way ANOVA with Bonferroni correction. [file Image_4.TIF]

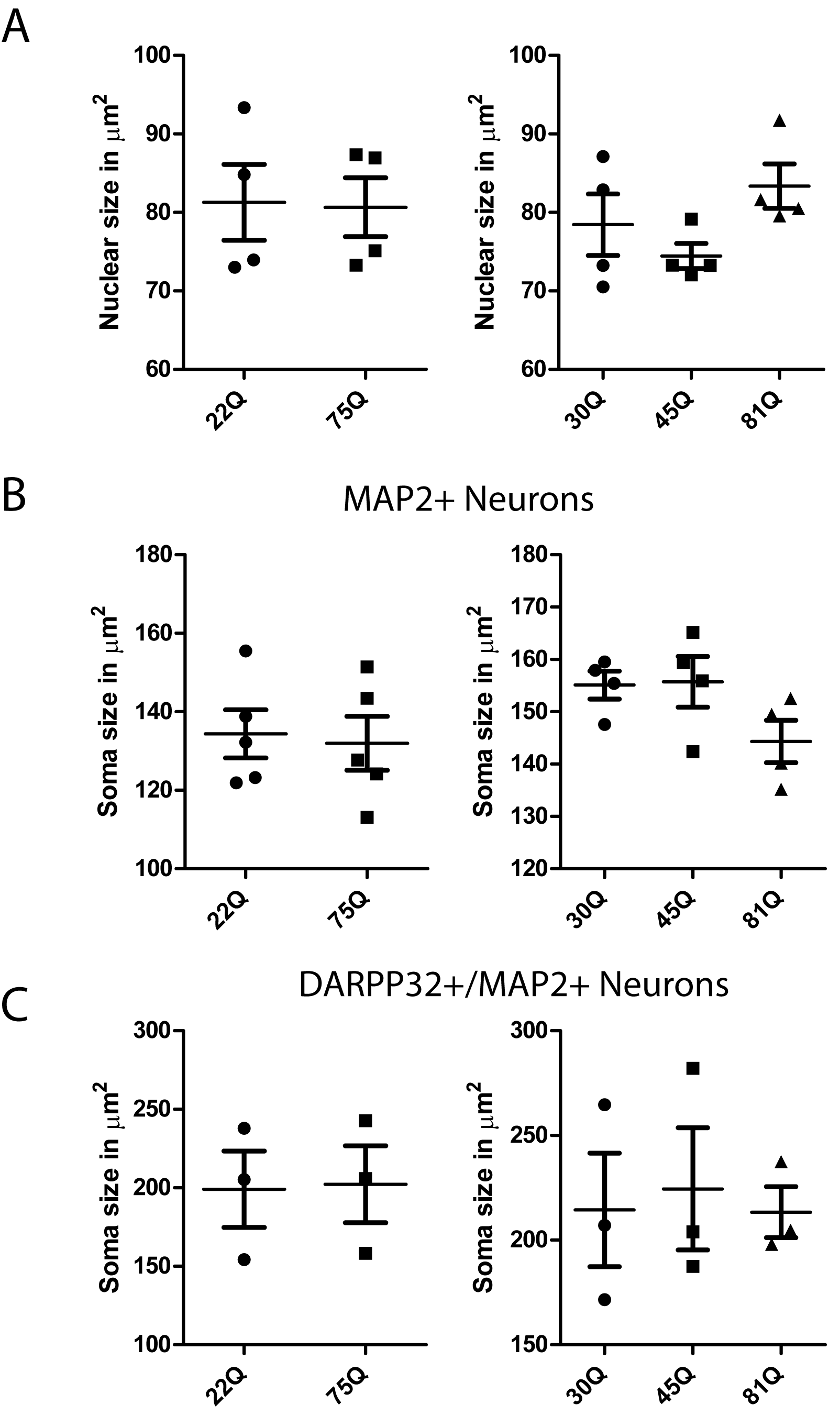

Supplement: Supplementary Figure 5 — Huntington’s disease PSC-derived medium spiny neuron cultures exhibit no overt morphological abnormalities. (A) Nuclear size was measured in neurons stained for MAP2 and Hoechst and no significant changes were detected between control and HD neurons in Family or IsoHD lines. (B) Cytoplasmic size of MAP2 + neurons also showed no significant changes between control and HD. (C) No significant changes were detected in the cytoplasm size of DARPP32 + /MAP2 + neurons. [file Image_5.TIF]

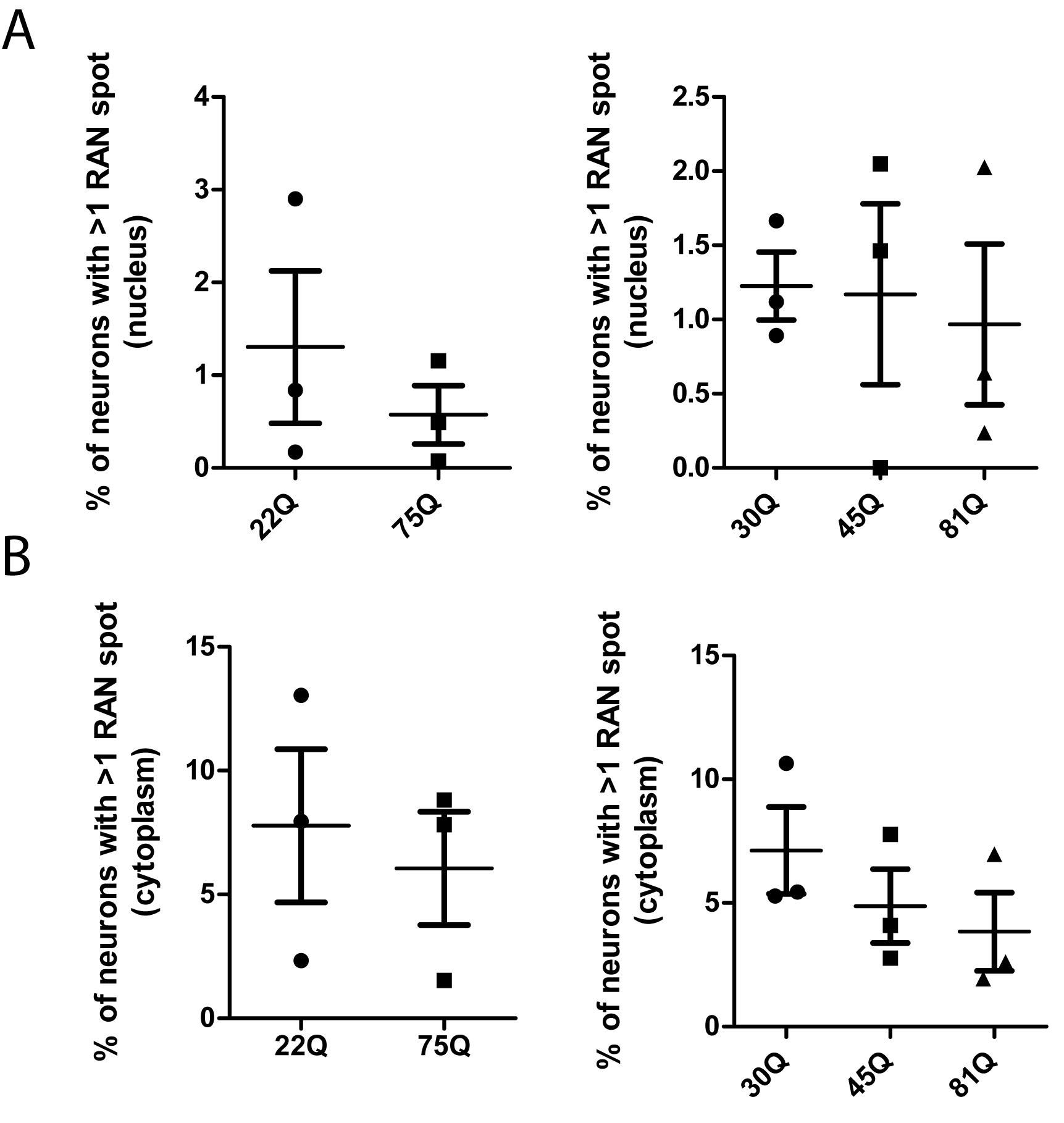

Supplement: Supplementary Figure 6 — Huntington’s disease PSC-derived medium spiny neuron cultures show no evidence of RAN aggregation. MAP2 + neurons stained for RAN exhibited RAN positive puncta, however the percentage of neurons with more than one RAN positive puncta in the nucleus (A) or cytoplasm (B) did not differ significantly between control and HD neurons in either Family HD or IsoHD lines. [file Image_6.TIF]

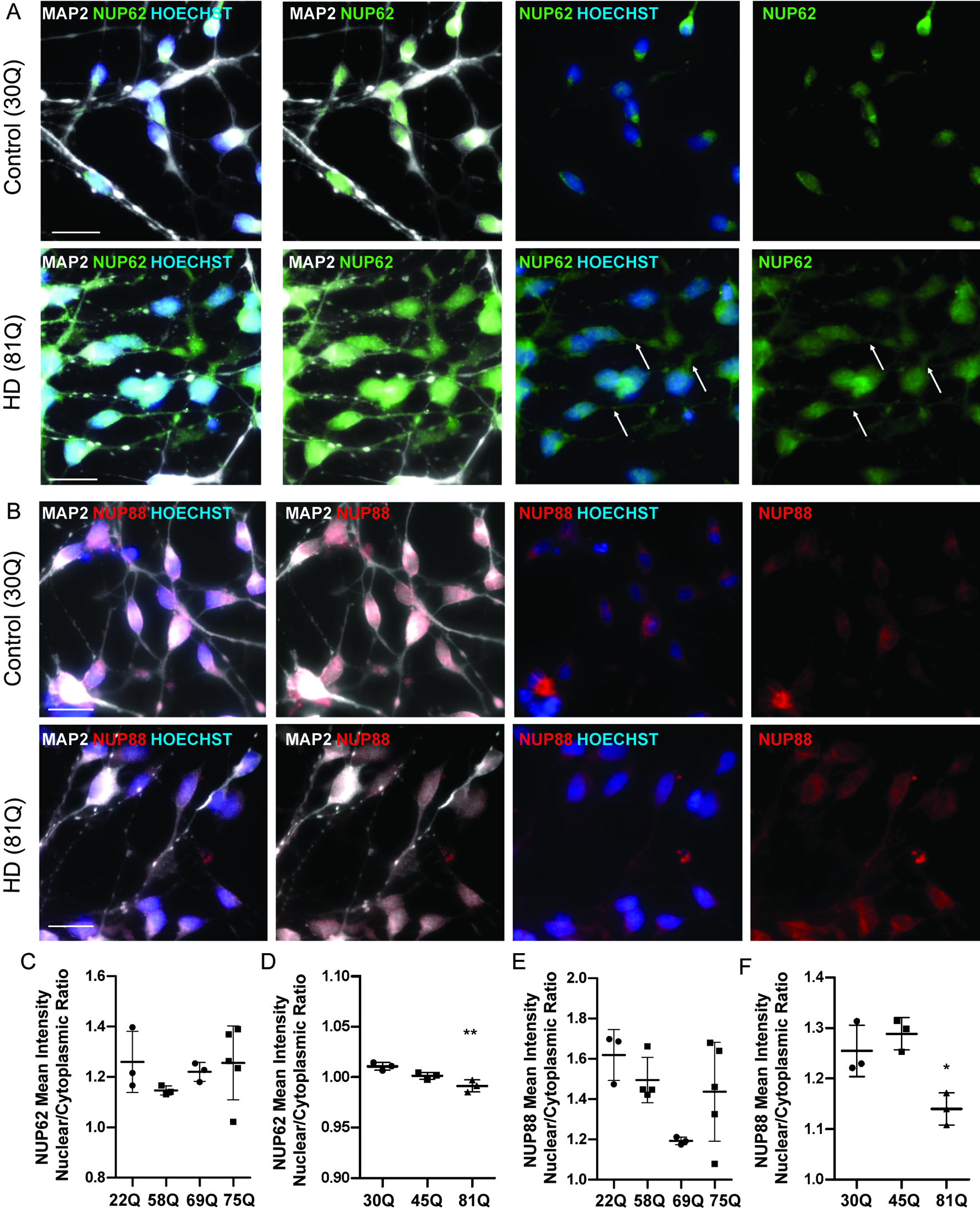

Supplement: Supplementary Figure 7 — Nucleoporins are mislocated in isogenic, but not Family line HD PSC-derived striatal neuronal cultures. (A) Neuronal cultures were stained with MAP2 and nucleoporins (NUP) 62 and (B) NUP88. Scale bar = 20 μM. (C) No significant differences were detected in the nuclear/cytoplasmic (N/C) ratio of NUP62 for the HD Family lines (58Q, 69Q, and 75Q). The N/C ratio of NUP62 was significantly lower in IsoHD 81Q neurons. (D) No significant differences were detected in the N/C ratio of NUP88 for the HD Family lines (58Q, 69Q, and 75Q). IsoHD 81Q neurons exhibited a significantly lower N/C ratio for NUP88, but not IsoHD 45Q neurons. *: p < 0.05, **: p < 0.01, ****: p < 0.0001. Data are presented as mean ± SEM of at least one differentiation of each clone of the HD Family iPSC lines, or three differentiations of each of the IsoHD ESC lines, analyzed by one-way ANOVA with Bonferroni correction. [file Image_7.TIF]

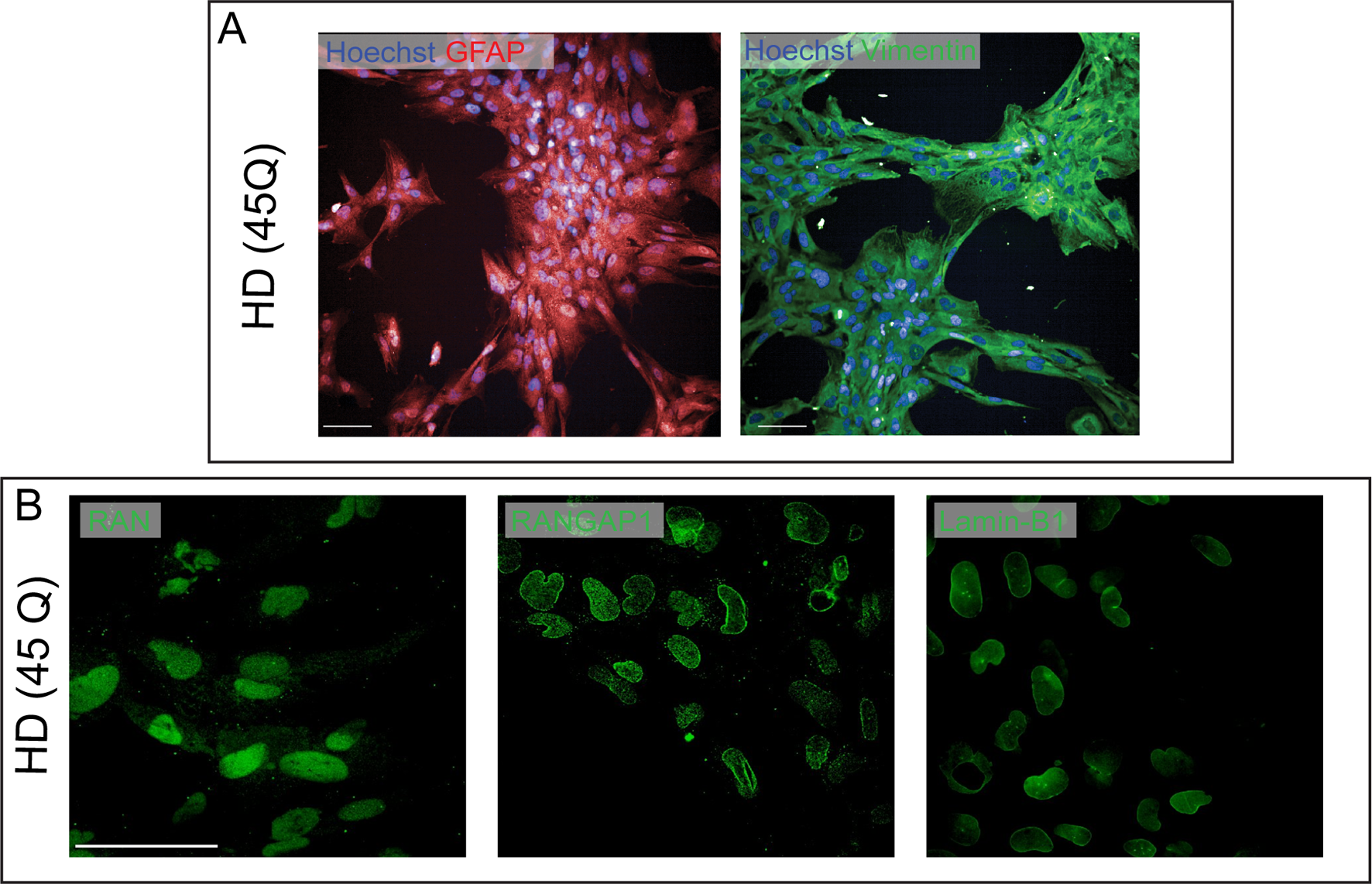

Supplement: Supplementary Figure 8 — Immunostaining Huntington’s disease PSC-derived astrocytes (45Q) for astrocyte markers and nuclear pore proteins. (A) IsoHD 45Q astrocytes stained positively for intermediate filament markers GFAP and Vimentin, confirming astrocyte identity. (B) IsoHD 45Q astrocytes were stained for RAN, RANGAP1 and lamin-B1 to investigate nuclear pore transport deficits. [file Image_8.TIF]

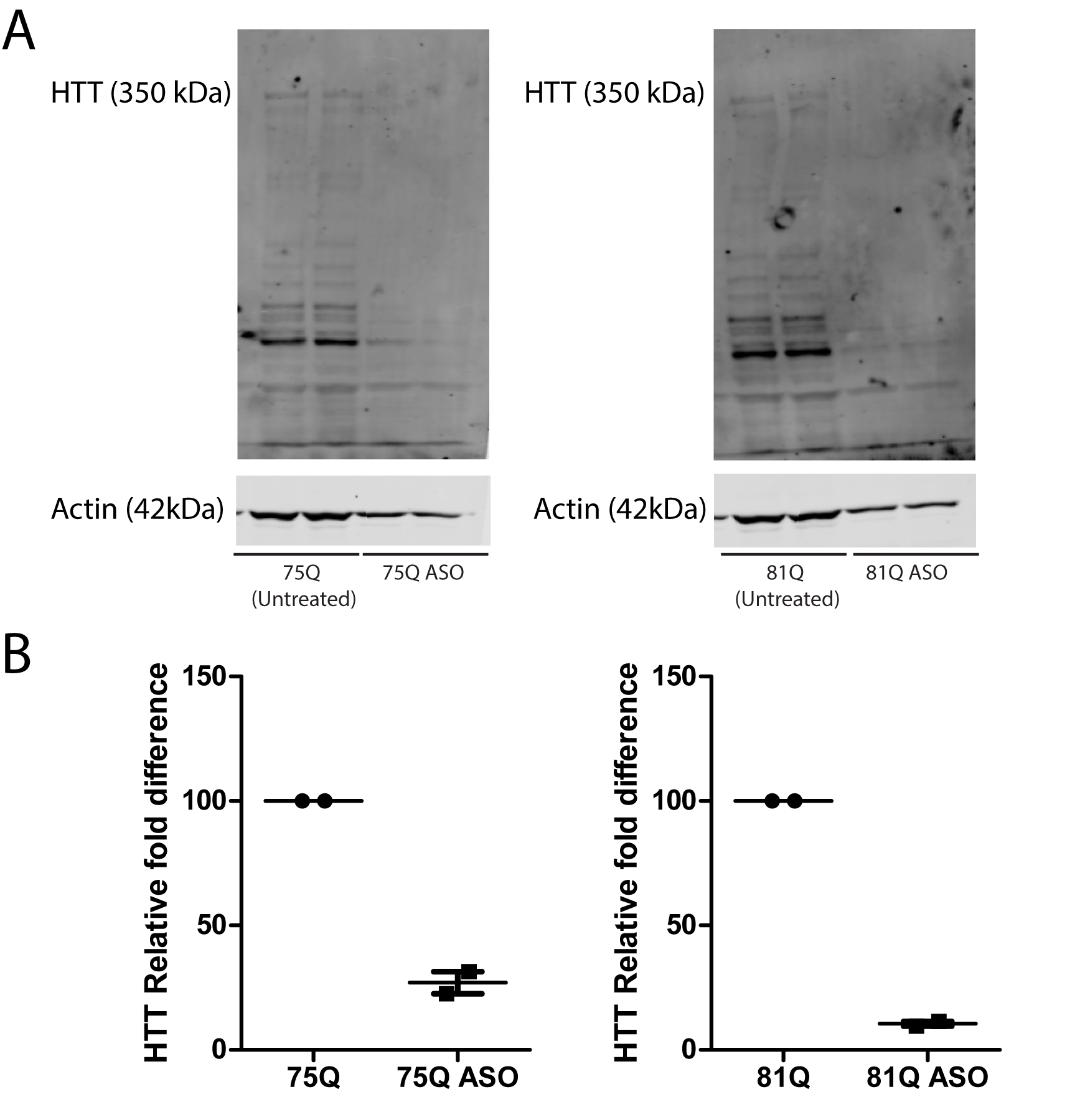

Supplement: Supplementary Figure 9 — Antisense oligonucleotide treatment successfully lowers huntingtin (HTT) protein levels in HD neurons. (A) Western blot of HTT and loading control Actin in untreated and ASO treated HD Family 75Q and IsoHD 81Q neuronal cultures at day 37. ASO treatment reduced full length HTT (350 kDa) as well as lower molecular weight fractions. (B) Full length HTT expression was normalized to Actin and measured. [file Image_9.TIF]

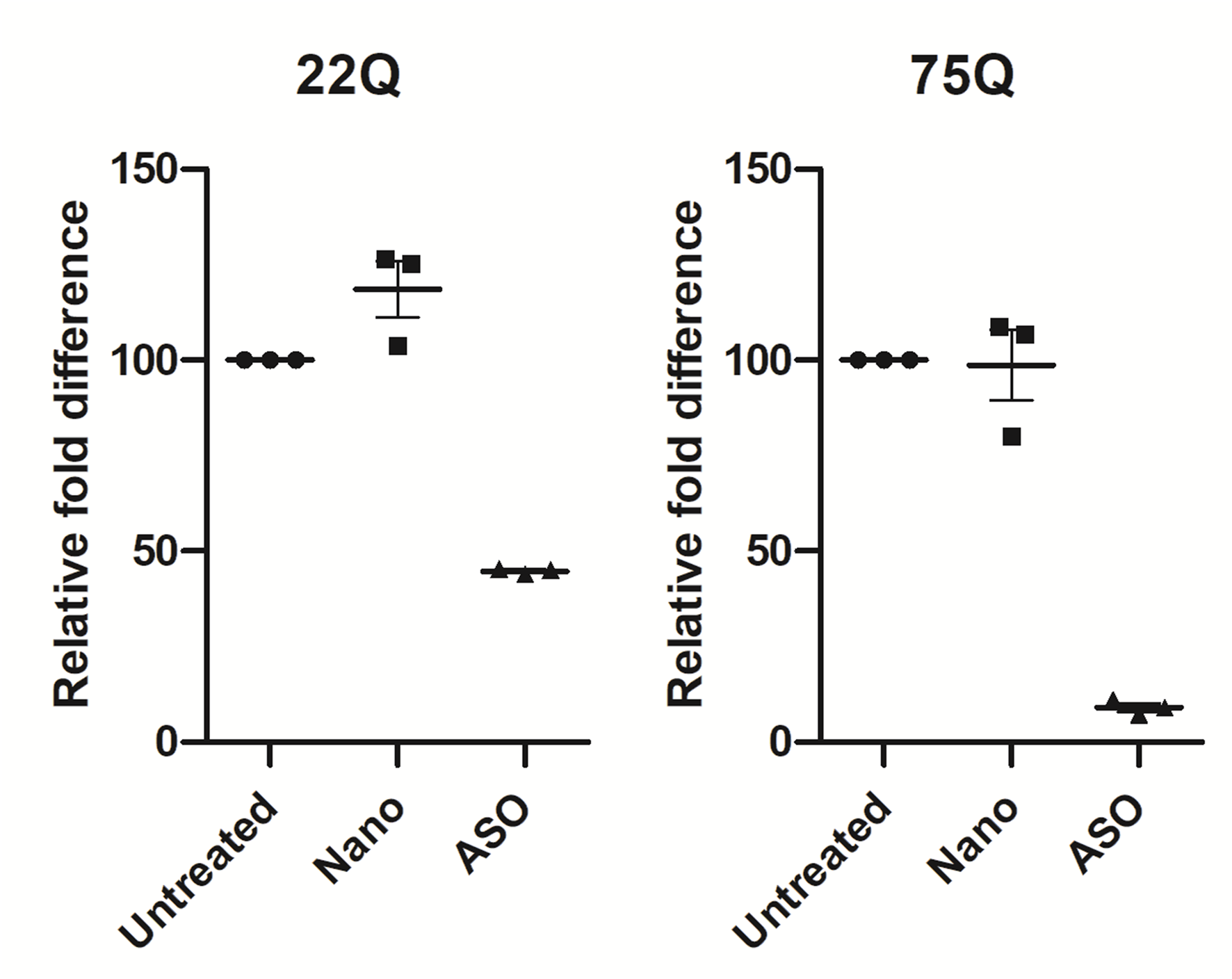

Supplement: Supplementary Figure 10 — Treatment with Nanoparticles (vehicle) has no effect on huntingtin (HTT) transcript levels. QPCR confirmed that treatment with nanoparticles only did not lower HTT in control (22Q) or HD (75Q) neurons. Values were normalized to housekeeping genes UBC, ATP5B and EIF4A. [file Image_10.TIF]

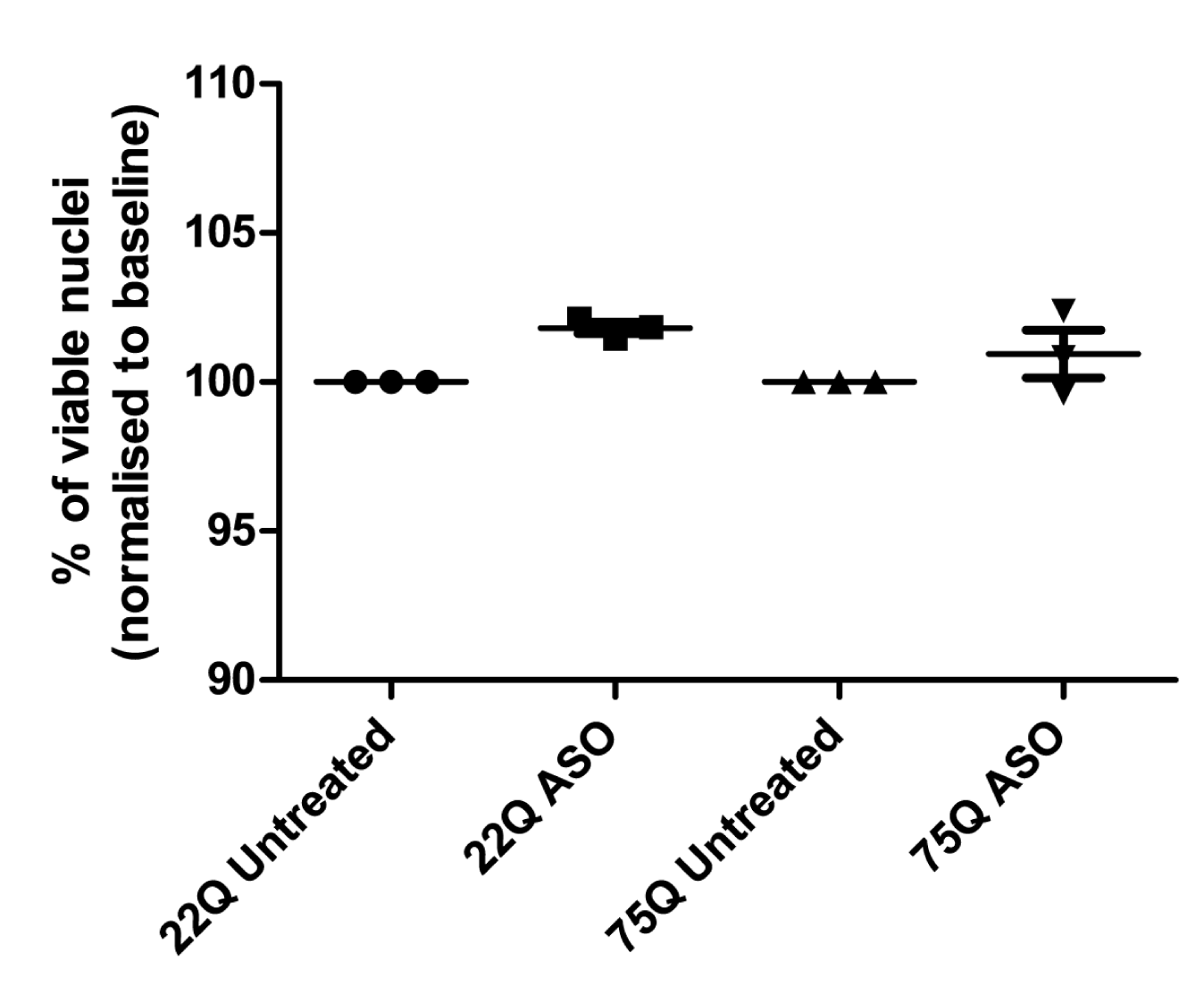

Supplement: Supplementary Figure 11 — Treatment with antisense oligonucleotides (ASO) to huntingtin (HTT) does not affect viability of neuronal cultures. The percentage of viable nuclei was determined by quantifying pyknotic or dysmorphic nuclei as an inverse proportion of the total number of cells. ASO treated cultures were normalized to their untreated counterparts and we did not detect any adverse effects of ASO treatment on neuronal viability. Data are presented as mean ± SEM of three clones of 22Q and 75Q, analyzed by one-way ANOVA with Bonferroni correction. [file Image_11.TIF]

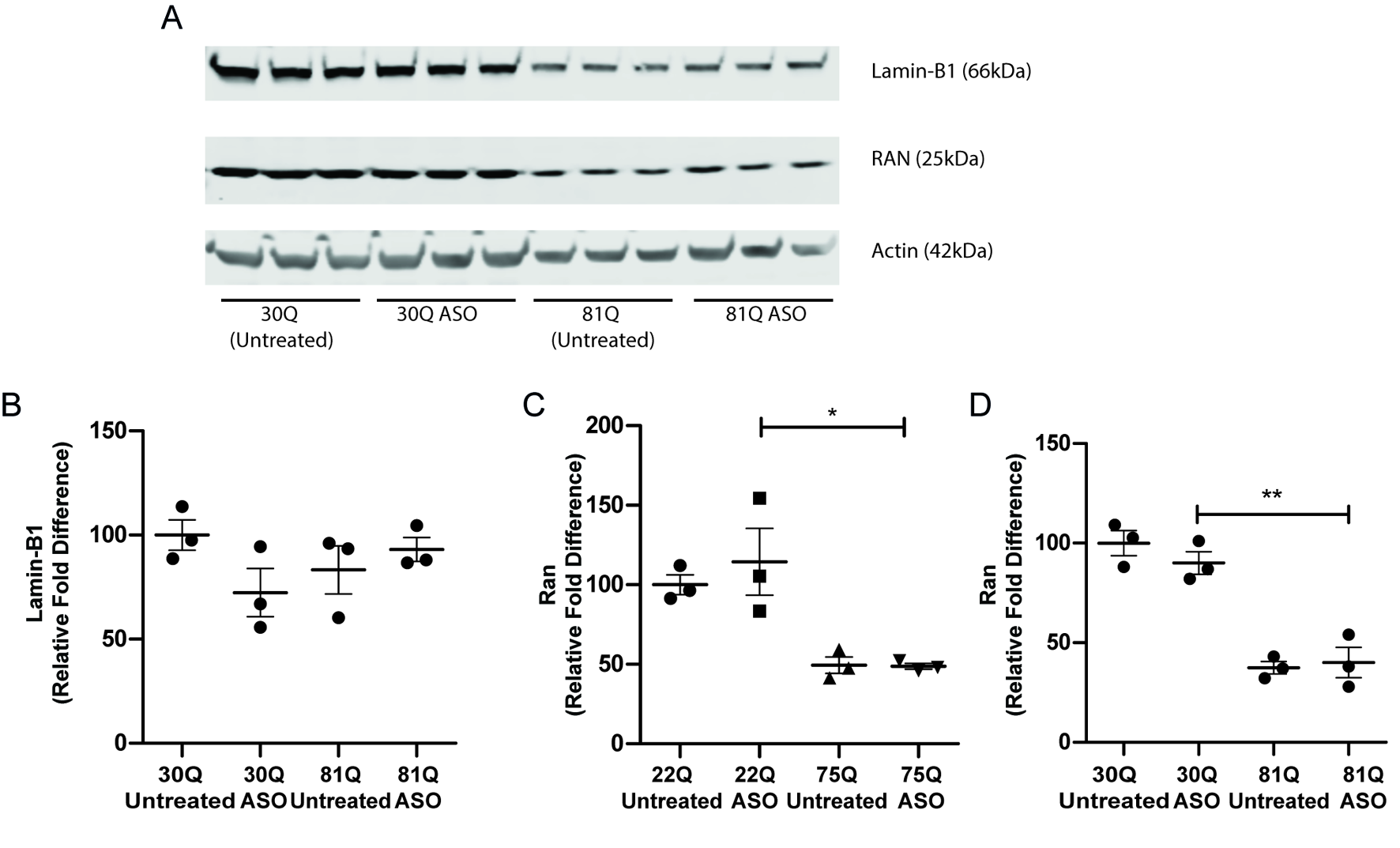

Supplement: Supplementary Figure 12 — Treatment with antisense oligonucleotides (ASO) to huntingtin (HTT) does not affect lamin-B1 and RAN protein expression in HD neurons. (A) Western blot of lamin-B1, RAN and loading control Actin in untreated and ASO treated IsoHD 30Q and 81Q neuronal cultures at day 37. (B) ASO treatment had no significant impact on lamin-B1 protein levels in IsoHD 30Q and 81Q neurons (C,D). No significant differences were detected in RAN protein expression in HD Family line control 22Q and 75Q cultures or IsoHD control 30Q and 81Q neuronal cultures. [file Image_12.TIF]

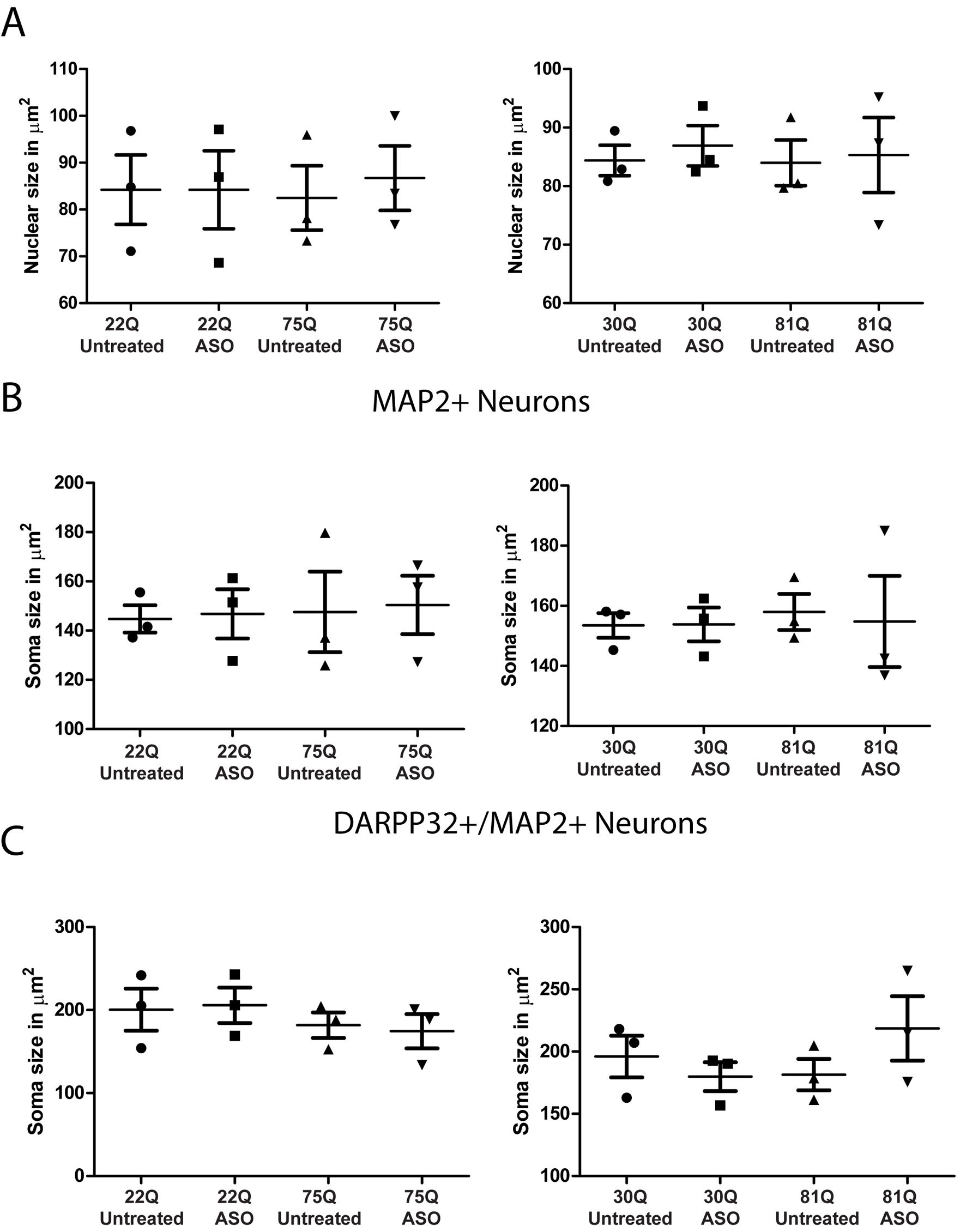

Supplement: Supplementary Figure 13 — Treatment with antisense oligonucleotides (ASO) to huntingtin (HTT) does not affect neuronal morphology. (A) Nuclear size and (B) cytoplasmic size of MAP2 + neurons, as well (C) cytoplasmic size of DARP32 + /MAP2 + neurons were not altered by ASO treatment. [file Image_13.TIF]
