# Supplementary material for: Mislocalization of Nucleocytoplasmic Transport Proteins in Human Huntington’s Disease PSC-Derived Striatal Neurons
Source: Front Cell Neurosci. 2021 Sep 29;15:742763. doi: 10.3389/fncel.2021.742763 (PMC8519404; doi:10.3389/fncel.2021.742763)

Supplemental methods

High Content Imaging analysis

Columbus image software (v2.8.0, Perkin Elmer) was used to analyse and quantify images by creating a script for each condition on the relevant control line (IsoHD 30Q or 22Q). We have provided details for the initial analysis below in addition to Table 4 and 5.

All analysis was completed within the parameters of the Columbus software using Columbus building blocks, which we then optimized to neuronal cultures (or astrocytes where applicable).

The first building block was always ‘Find Nuclei’. For neuronal cultures we used ‘Method M’ to detect HOECHST33342 positive nuclei, specifying a diameter of 15 µm, splitting coefficient 0.4 and common threshold 0.1. We found that these parameters selected the majority of nuclei in our cultures as shown in image below.
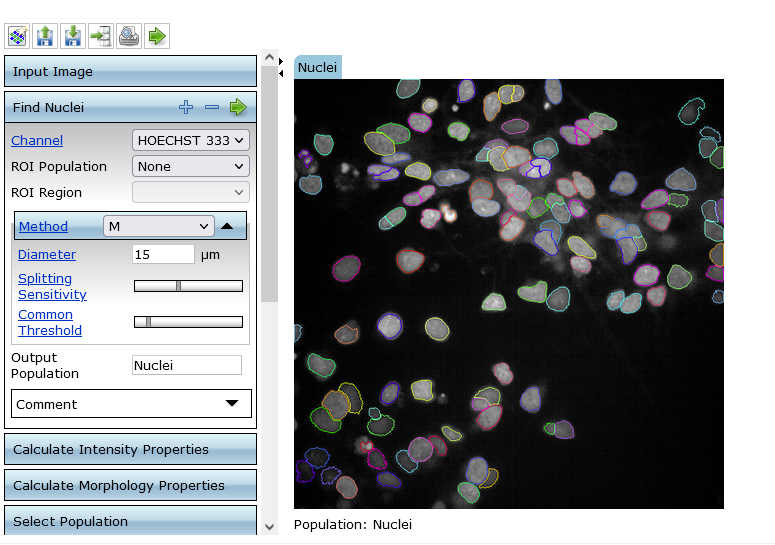
.

We next calculated ‘Intensity Properties’ and ‘Morphological Properties’ for HOECHST 33342 positive nuclei using standard Columbus parameters without changes. We then used these values to exclude pyknotic or dysmorphic nuclei by restricting Nucleus size to above 40 µm^2^. We also excluded clusters of nuclei that may be grouped together by excluding nuclei with a size above 250 µm^2^. Nuclei with a HOECHST 33342 mean intensity >18500 were also excluded as the excessively bright staining indicates apoptotic or necrotic cells. Boolean operations (under output population) were left unchanged, according to Columbus parameters. Example image shown below. Green nuclei were considered viable nuclei, red nuclei were excluded from the analysis.


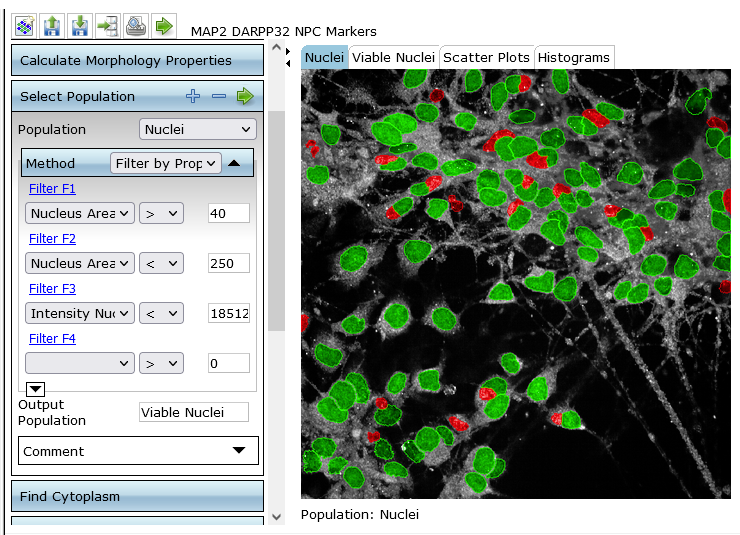


Our next building block was the ‘Find Cytoplasm’ function. For neurons we selected Method B (Common threshold 0.45, Individual threshold 0.15) in channel Alexa 633 which we used for the MAP2 or BIII-tubulin stain. We found that these parameters successfully detected cytoplasmic areas, including some neurites. Example image shown below. Parameters were adjusted for astrocytes and Method A (Individual threshold 0.15) was used to detect the cytoplasm.
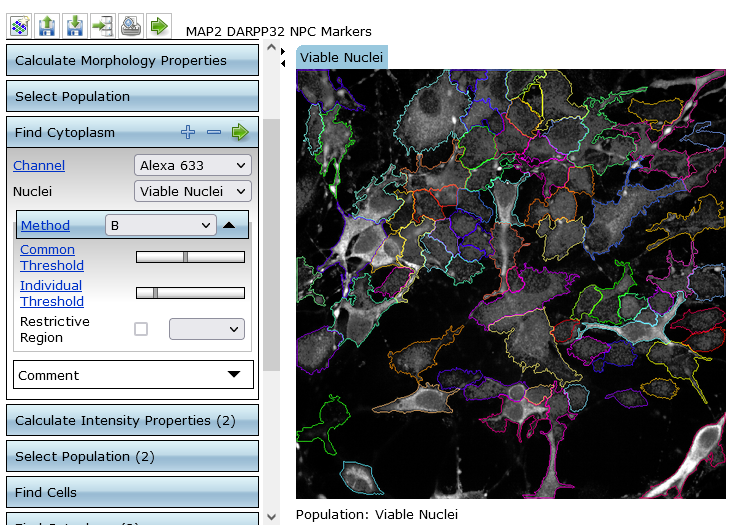


Once nuclear and cytoplasmic areas have been defined, these can then be used to calculate staining intensities in other channels, which can be used to define populations (eg DARPP32 positive neurons). We used nuclear and cytoplasmic staining intensities of RAN, RANGAP1 and lamin-B1 to obtain nuclear/cytoplasmic ratios. Example image below shows Map2+ stained neurons (in grey) with DARPP32+ neurons selected and cells partitioned into nuclei and cytoplasm to obtain cytoplasmic Intensities for RAN.


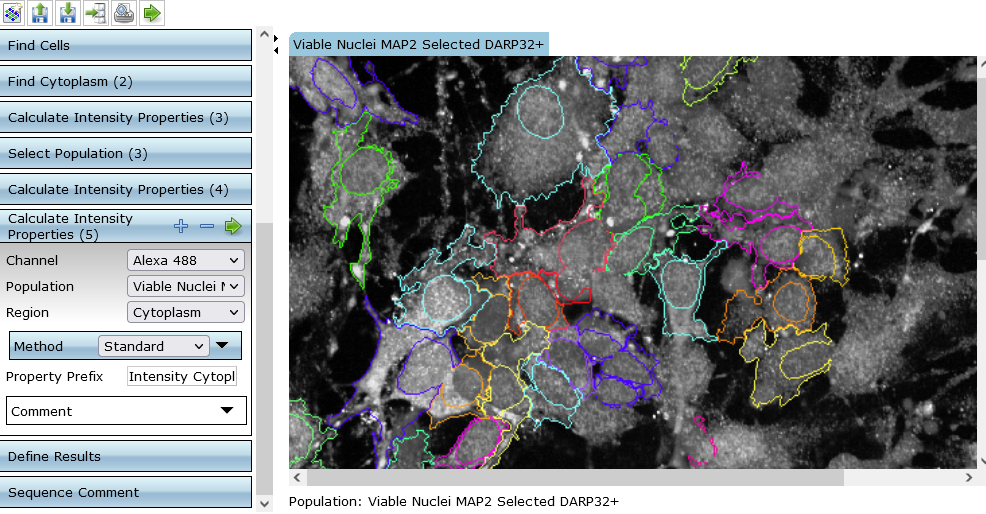


For astrocytes, the first building block was also ‘Find nuclei’ and we selected Method B (Diameter >30 µm, common threshold 0.4) to account for larger nucleus and cell size. These parameters successully selected the majority of nuclei.
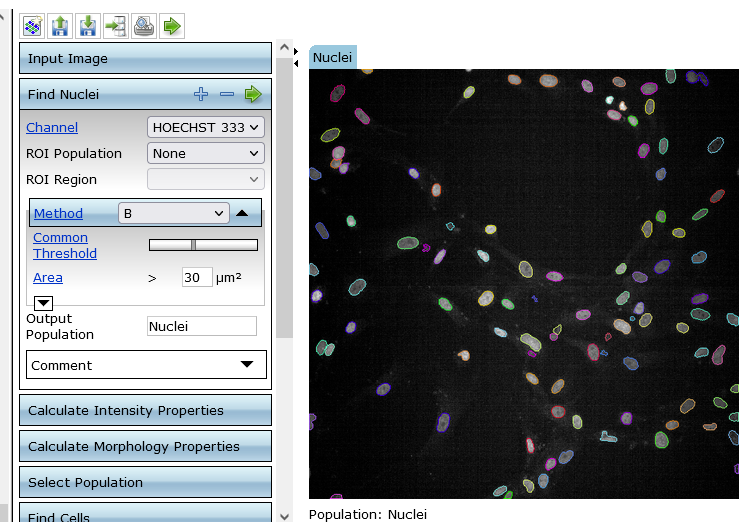


As with neuronal cultures, we next calculated ‘Intensity Properties’ and ‘Morphological Properties’ for HOECHST 33342 positive nuclei using standard Columbus parameters without changes. These values were again used to exclude pyknotic or dysmorphic nuclei by restricting Nucleus size to above 40 µm^2^. Due to the large nucleus size of astrocyte, we excluded nuclei with a size larger than 1000 µm^2^. Nuclei with a HOECHST 33342 mean intensity >18500 were also excluded as the excessively bright staining indicates apoptotic or necrotic cells, as well as nuclei with roundness <0.4. Boolean operations (under output population) were left unchanged, according to Columbus parameters. Example image shown below. Green nuclei were considered viable nuclei, red nuclei were excluded from the analysis.
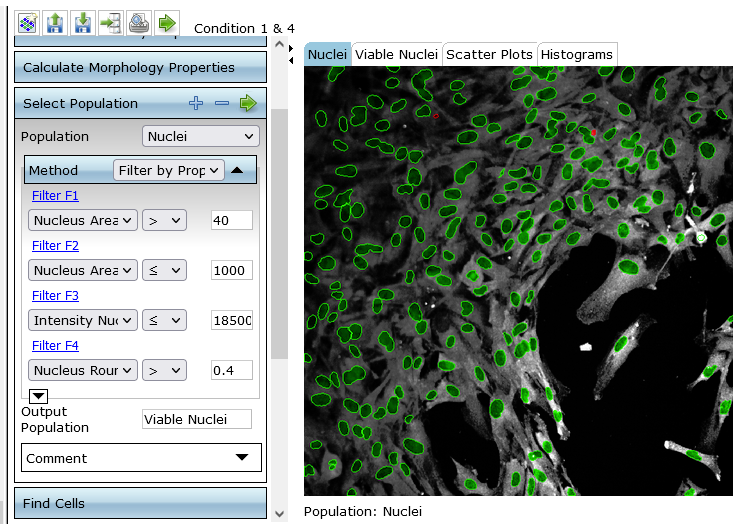


The next building block was the ‘Find Cytoplasm’ function. GFAP (channel 633) or Vimentin (channel 488) were used to visually the cytoplasm of astrocytes. Method A (Individual threshold 0.15) was used to detect the cytoplasm on Columbus which successfully separated cytoplasmic areas of very dense cultures. Example image shown below.
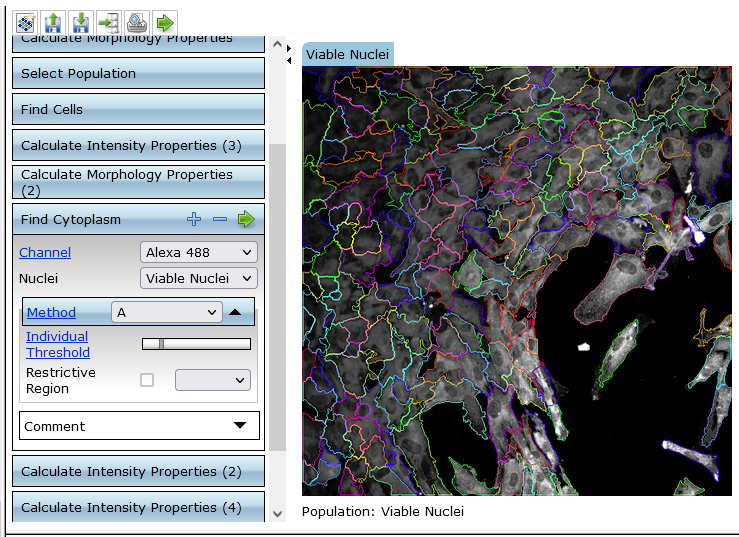


Once nuclear and cytoplasmic areas have been defined, these can then be used to calculate staining intensities in other channels. We used nuclear and cytoplasmic staining intensities of RAN, RANGAP1 and lamin-B1 to obtain nuclear/cytoplasmic ratios. Example image below shows Vimentin+ stained astrocytes (in grey) with cells partitioned into nuclei and cytoplasm to obtain cytoplasmic Intensities for RAN.


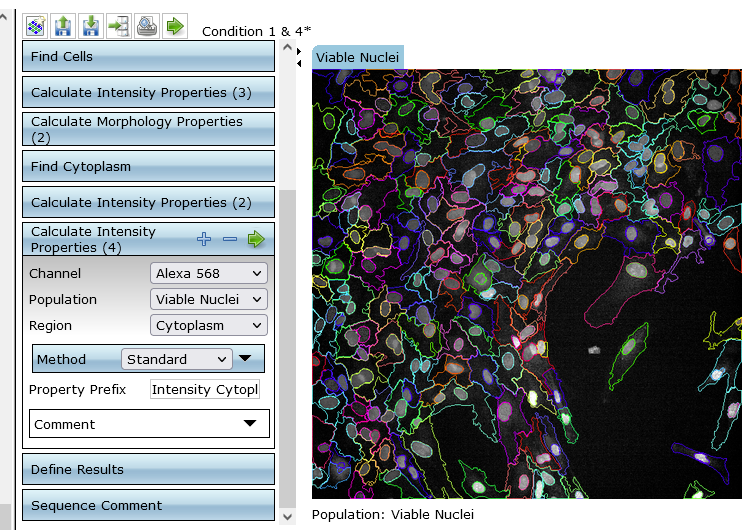

Supplement: Supplementary file 14 [file Data_Sheet_1.docx]
